# Effect of scavenging on predation in a food web

**DOI:** 10.1002/ece3.7525

**Published:** 2021-05-03

**Authors:** Jarad P. Mellard, Sandra Hamel, John‐André Henden, Rolf A. Ims, Audun Stien, Nigel Yoccoz

**Affiliations:** ^1^ Department of Arctic and Marine Biology UiT The Arctic University of Norway Tromsø Norway; ^2^ Département de biologie Université Laval Québec Canada

**Keywords:** feedbacks, food web, predation, scavenging

## Abstract

Scavenging can have important consequences for food web dynamics, for example, it may support additional consumer species and affect predation on live prey. Still, few food web models include scavenging. We develop a dynamic model that includes two facultative scavenger species, which we refer to as the predator or scavenger species according to their natural scavenging propensity, as well as live prey, and a carrion pool to show ramifications of scavenging for predation in simple food webs. Our modeling suggests that the presence of scavengers can both increase and decrease predator kill rates and overall predation in model food webs and the impact varies (in magnitude and direction) with context. In particular, we explore the impact of the amount of dynamics (exploitative competition) allowed in the predator, scavenger, and prey populations as well as the direction and magnitude of interference competition between predators and scavengers. One fundamental prediction is that scavengers most likely increase predator kill rates, especially if there are exploitative feedback effects on the prey or carrion resources like is normally observed in natural systems. Scavengers only have minimal effects on predator kill rate when predator, scavenger, and prey abundances are kept constant by management. In such controlled systems, interference competition can greatly affect the interactions in contrast to more natural systems, with an increase in interference competition leading to a decrease in predator kill rate. Our study adds to studies that show that the presence of predators affects scavenger behavior, vital rates, and food web structure, by showing that scavengers impact predator kill rates through multiple mechanisms, and therefore indicating that scavenging and predation patterns are tightly intertwined. We provide a road map to the different theoretical outcomes and their support from different empirical studies on vertebrate guilds to provide guidance in wildlife management.

## Graphical abstract

Scavenging, while often ignored, can have major impacts on predation and food web dynamics. We convert empirical observations on scavenging and predation into a theoretical framework to explain observed contrasting patterns and make predictions across different food webs.

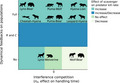

## 1 INTRODUCTION

Scavenging, or the use of carrion for energy gain, is an important energetic pathway in food webs. Some species are specialized scavengers, but most vertebrate predators also operate as facultative scavengers by returning to scavenge their own kills or kills of others (Moleón et al., 2014). Predator‐killed prey can be the most significant source of biomass for scavengers in some ecosystems (Elbroch & Wittmer, 2012; Wikenros et al., 2013). This, along with other recent evidence (Andrén et al., 2011; Krofel et al., 2012; Tallian et al., 2017), suggests a strong interaction between scavenging and predation. However, while predation has been a core subject in ecological research for decades, scavenging in a food web context has not received the theoretical or empirical attention it deserves (Moleón & Sánchez‐Zapata, 2015). This has led to recent calls for more focus on the link between predation and scavenging (Moleón et al., 2014; Wilson & Wolkovich, 2011).

Scavenging can impact predation in multiple ways. High availability of carcasses is likely to decrease kill rates by predators that are facultative scavengers. However, the presence of other scavenger species may increase predation rates as kills of predators get consumed by others (Andrén et al., 2011). Currently, there are conflicting ideas and varying reports on how scavenging affects predation in different vertebrate predator guilds (Allen et al., 2015; Krofel et al., 2012). In systems with wolves and bears, in both Yellowstone National Park and Scandinavia, the focal predator, the wolf, seems to kill less when scavenging brown bears are present (Tallian et al., 2017). In contrast, in the mountains of Slovenia and Croatia, lynx increase predation rates in the presence of brown bears (Krofel et al., 2012). Thus, the species of predator and scavenger seems to matter. Moreover, scavenging the kills of other species is mostly asymmetrical in food webs, with one species more likely to scavenge another species kills than vice versa (Allen et al., 2015; Krofel et al., 2012).

In a model of lynx and wolverines, Andrén et al. (2011) found that for a given abundance of lynx and wolverines, scavenging by wolverines reduced total predation. However, predation strategies and densities of both the predators and scavengers were kept constant, without the dynamical feedbacks in strategies or densities expected in natural systems. Thus, whether total predation and other predation metrics increase or decrease in natural systems remains uncertain. The answer to this question, however, would be highly beneficial for wildlife management and conservation that often have to take unpopular or controversial management decisions regarding predators and scavengers (Hunter et al., 2018; Serrouya et al., 2019; Walsh et al., 2012). We therefore addressed this question, taking aim at providing a road map of the theoretical outcomes and the empirical support. We build on previous work examining the interaction of predators and scavengers by creating dynamic models to address how predation rates change with respect to densities of prey and carrion and how changes in predator/scavenger population densities affect these measures. Specifically, we are interested in predation by the main predator, if their kill rates increase or decrease when a scavenger is added to the food web.

We build a generalized model that can be applied to different case studies, focusing on different combinations of two interacting species of predators/scavengers from different habitats around the world. We want to understand how the addition and increasing abundance of a scavenger to a food web affects carrion dynamics, kill rates of the primary predator, and concomitant losses of the prey species. We consider a food web with two mammalian facultative scavenger species, one which we refer to as the focal predator species (with a propensity for predation), and the other which we refer to as the focal scavenger species (with a propensity for scavenging), since most scavengers are facultative (Moleón et al., 2014).

Many mammalian predator/scavenger populations are controlled by management to low numbers (Prugh et al., 2009; Reynolds & Tapper, 1996; Treves & Karanth, 2003), which may prevent many of the natural feedbacks in population growth from occurring and has major impacts on other species and the ecosystem (Estes et al., 2011; Ripple et al., 2014). Even when predator and scavenger populations are controlled, prey and carrion are likely to have coupled dynamics as they are consumed. We use different constrained versions of the model to represent different natural and managed systems in order to understand how variation in dynamic feedbacks (exploitative competition) affects scavenging and predation patterns in these systems. In addition, we investigate how the direction and magnitude of interference competition between the predator and scavenger affect predation rates. Thus, our models not only cover the different assumptions of feedbacks and population regulation, but also include species interactions of both exploitative competition and direct interference competition known to occur in ecological food webs (Krofel et al., 2012; Mattisson et al., 2011; Tallian et al., 2017).

We begin by dissecting the question of why an animal would scavenge and then discuss it as an adaptive strategy, which we build into a mathematical model. We then build that model into a model of kill rates, but that has no population dynamics. Finally, this is converted into a fully dynamical model of a food web with feedbacks, including population dynamics. In our analyses, we take the approach of using several simplified models and assumptions, which can be relaxed as shown in the Appendix A. We focus on our central question of how predation is affected by the addition of a scavenger to the food web.

## 2 BUILDING A MODEL OF SCAVENGING

We develop a general model based on optimal foraging theory that allows changes in the strategies and densities of predators/scavengers in response to population changes in prey and carcass availability.

### 2.1 Profitability of scavenging

We use this section to describe why it is likely an animal would initially try to scavenge a carcass if one is available. We assume carrion to be more profitable than live prey, following previous studies, for example, Moleón et al. (2015). However, we found surprisingly little information in the literature on why it is profitable to scavenge so we think it is important to illustrate with this profitability model that some basic information is lacking in ecological systems that involve scavenging (Moleón et al., 2014; Wilson & Wolkovich, 2011).

Profitability depends on handling time, searching time, and energy content, along with mobility of prey (but see Sih and Christensen (2001) on why it may be hard to compare mobile and immobile prey). For example, equation 1 in Schoener (1971) has for food item *i*.

(1) $$\frac{e_{i}}{t_{i}}=\frac{\text{potential energy}‐\text{pursuit costs}‐\text{handling and eating costs}}{\text{pursuit time}+\text{handling and eating time}}.$$

Thus, it makes sense for many animals to investigate a carcass to see if enough energy content could be foraged from it. Energy content of a carcass can be less than a live prey item but often there is still some unknown amount of energy remaining. However, the handling time of a carcass versus live prey item can easily make up for the difference in energy content. In the systems we describe, the profitability of scavenging typically will be higher than predation given the high handling time associated with killing large prey.

We are building on previous optimal diet theory (Abrams & Matsuda, 2004; Charnov, 1976; Fryxell & Lundberg, 1994; MacArthur & Pianka, 1966; Schoener, 1971) where decisions are made to attack based on initial or likely energy and handling time. Animals may not forage optimally and may not have perfect information (Sih & Christensen, 2001). This may also be the case with carrion, that is, they do not know how much of a carcass remains but will approach it assuming it could have a large portion of energy content remaining.

### 2.2 Adaptive behavior of predator

According to Fryxell and Lundberg (1994), predator diet should be a sigmoid function of the density of the most profitable prey, where profitability is defined as

$$\text{Profitability}=\frac{\text{energy content}}{\text{handling time}}=\frac{e}{h}$$

following MacArthur and Pianka (1966) and Charnov (1976).

We assume carrion to be more profitable than live prey (Moleón et al., 2015). Formalizing this, e/h for carrion > e/h for live prey. Therefore, we make the scavenging propensity s depends on carrion density C,

(2) $$s\left(C\right)=\frac{zC^{b}}{1+zhC^{b}}$$

where z and b are scaling coefficients that change the magnitude and shape of the scavenging response to carrion density, effectively controlling the switching response. Many theoretical studies of adaptive foraging include a similar formulation (Abrams & Matsuda, 2004; Charnov, 1976; Fryxell & Lundberg, 1994; MacArthur & Pianka, 1966).

**FIGURE 1:**
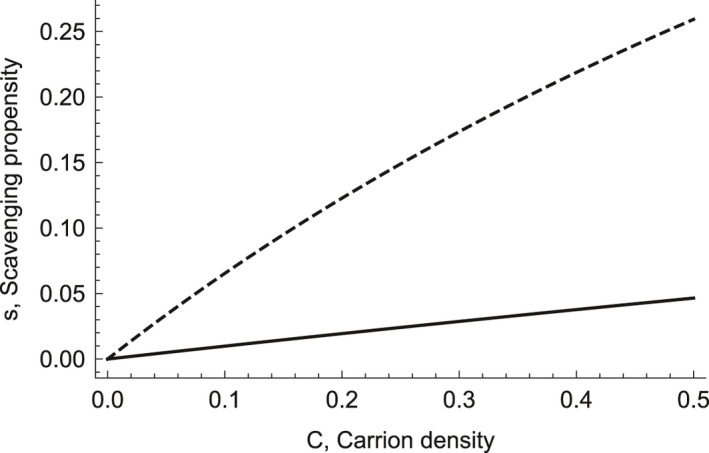
Scavenging propensity with carrion density for the predator (solid line) and scavenger (dashed line) both of whom are facultative scavengers. Note that resultant scavenging rates are also a function of prey availability and competition for prey (see further for a model that builds on this natural scavenging propensity). Parameters (handling time, h, and scaling coefficients of the scavenging response to carrion density, z and b) for the predator P and scavenger S with corresponding subscripts are as follows: $h_{\mathrm{CP}}=1.5;h_{\mathrm{CS}}=1;b_{P}=0.1;b_{S}=1;z_{P}=0.1;z_{S}=0.7$

We consider two facultative scavenging species which we refer to as the focal predator and scavenger, because we assume they have different scavenging propensities (Figure 1). If the attack propensity on live prey f is negatively related to scavenging propensity s, then the predator, with a lower scavenging propensity, will be more specialized on the live prey over most carrion densities, while the scavenger, with a higher scavenging propensity, will be more specialized on the carrion over most carrion densities. This is a realistic trade‐off for many predator and scavenger pairs (Krofel et al., 2012) and can also be related to the handling time of carrion—for example, a scavenger such as the wolverine can have a lower handling time than a predator such as the lynx because the lynx opens the carcass, a kind of facilitation (Kane et al., 2017).

### 2.3 Calculation of predation

The equations for kill rates and scavenging rates are a form of the multispecies disk equation (Charnov, 1976; Fryxell & Lundberg, 1994). The “disk” equation was developed (Holling, 1959) to describe a saturating functional response of predators attacking prey that takes into account handling time. Kill rates $k_{P}$ and $k_{S}$ for the predator P and scavenger S respectively are

(3) $$k_{P}=\frac{f_{P}R}{1+f_{P}Rh_{\mathrm{RP}}+s_{P}Ch_{\mathrm{CP}}},$$

(4) $$k_{S}=\frac{f_{S}R}{1+f_{S}Rh_{\mathrm{RS}}+s_{S}Ch_{\mathrm{CS}}},$$

where R and C are the abundances of prey and carrion, respectively; $f_{P}$ and $f_{S}$ are the predation propensities of the predator and scavenger, respectively, on the prey; $s_{P}$ and $s_{S}$ are the scavenging propensities of the predator and scavenger, respectively, on the carrion; $h_{\mathrm{RP}}$ and $h_{\mathrm{RS}}$ are the handling times of the predator and scavenger, respectively, on the prey; $h_{\mathrm{CP}}$ and $h_{\mathrm{CS}}$ are the handling times of the predator P and scavenger S, respectively, on the carrion.

We allow the attack propensity on live prey $f_{i}$ and scavenging propensity $s_{i}$ to be flexible foraging strategies. However, one likely impacts the other, as is observed in wolverines for example (Mattisson et al., 2016). Thus, we tested whether defining a linear trade‐off between $f_{i}$ and $s_{i}$ impacted results and we found the same qualitative results whether we set $f_{i}$ to a constant value (Fryxell & Lundberg, 1994) or imposed the constraint that the attack propensity on live prey $f_{i}$ depends on scavenging propensity $s_{i}$, so that $f_{i}+s_{i}$ = constant for i=P or S.

The total number of prey killed by the predator and scavenger per time unit as defined by Equations (3) and (4) is

(5) $${\text{Kills}}_{\text{Total}}=k_{P}P+k_{S}S$$

where P and S are the abundances of the predator and scavenger, respectively. Results with respect to other metrics of predation such as per‐capita kill rates are presented in the Appendix A.

## 3 FULL FOOD WEB MODEL

**FIGURE 2:**
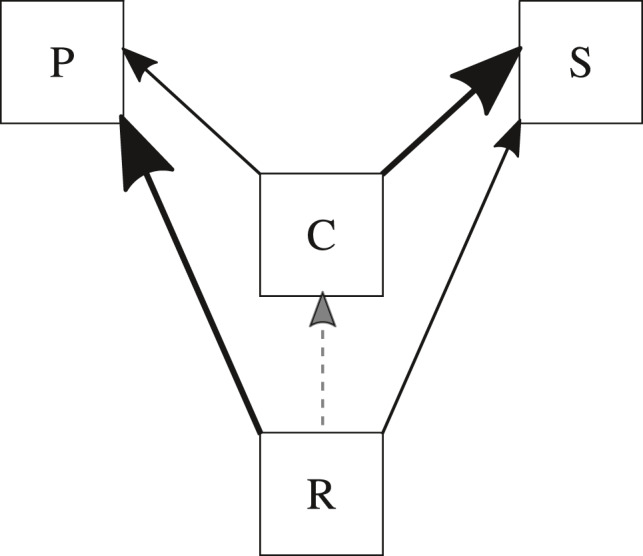
Full model with state variables for prey resource R, carrion C of prey killed by primary predators P and scavengers S, both of whom are facultative scavengers. Lines connecting state variable boxes represent potential energetic (biomass) pathways, with thickness of solid lines and size of arrows indicating relative specialization of the predator and scavenger on the two types of resources R and C, where dashed line indicates conversion of prey resource R to carrion C

The general model topology is depicted in Figure 2.

### 3.1 Full dynamical model equations

The full model for the prey R, carrion C, primary predator P, and scavenger S in continuous time (Focardi et al., 2017; O'Bryan et al., 2019) is given by.

(6) $$\frac{\mathrm{dR}}{\mathrm{dt}}=g\left(R\right)‐k_{P}P‐k_{S}S,$$

(7) $$\frac{\mathrm{dC}}{\mathrm{dt}}=\left(1‐\phi_{P}\right)k_{P}P+\left(1‐\phi_{S}\right)k_{S}S‐q_{P}P‐q_{S}S‐\delta C,$$

(8) $$\frac{\mathrm{dP}}{\mathrm{dt}}=P\left(‐m_{P}+\phi_{P}k_{P}a_{P}+q_{P}a_{P}\right),$$

(9) $$\frac{\mathrm{dS}}{\mathrm{dt}}=S\left(‐m_{S}+\phi_{S}k_{S}a_{S}+q_{s}a_{S}\right),$$

where $\phi_{P}$ and $\phi_{S}$ are the proportions of a killed prey immediately consumed by the predator P and scavenger S, respectively (Table 1), so $1‐\phi_{P}$ and $1‐\phi_{S}$ are the proportions of a killed prey immediately converted to carrion by the predator P and scavenger S, respectively; $m_{P}$ and $m_{S}$ are the mortality rates of the predator P and scavenger S, respectively; and $a_{P}$ and $a_{s}$ are conversion factors of prey or carrion to predator P and scavenger S densities, respectively.

**TABLE 1:** Parameter definitions and values used in analyses unless noted otherwise

Variable or parameter	Definition	Value (Range)
R	Prey resource population	State variable
*C*	Carrion	State variable
P	Predator population	State variable
S	Scavenger population	State variable
$h_{\mathrm{Ci}}$ [time^−1^]	Handling time of carrion by P or S for i=P or S	Function of S or P
$h_{\mathrm{Ri}}$ [time^−1^]	Handling time of prey by P or S for i=P or S	4 (1:8)
$n_{i}$ [Dimensionless]	Interference competition coefficient for P or S for i=P or S	0 (−1:1)
$y_{i}$ [Dimensionless]	Scaling coefficient for interference P or S for i=P or S	1 (0.1:2)
$\phi_{i}$ [Dimensionless]	Proportion immediately consumed by P or S for i=P or S	0.465 (0:1)
$f_{i}$ [time^−1^]	Predation propensity of P or S for i=P or S	Function or (0.1:1)
$s_{i}$ [time^−1^]	Scavenging propensity of P or S for i=P or S	Function of C
$b_{i}$ [Dimensionless]	Scaling coefficient for $s_{i}$ for i=P or S	1 (0:3)
$z_{i}$ [Dimensionless]	Scaling coefficient for $s_{i}$ for i=P or S	1 (0:1)
$m_{i}$ [time^−1^]	Mortality rate of P or S for i=P or S	0.1,0.21 (0.03:0.5)
$a_{i}$ [Dimensionless]	Conversion factor of P or S for i=P or S	1 (0.75:1)
δ [time^−1^]	Background loss rate of C due to other processes	0 (0:0.5)

Note

See also Appendix A Section A.3.1.

δ is the background loss rate of carrion due to other scavengers, decomposition, and the environment, and $g\left(R\right)$ is the input of the prey to the system defined as $g\left(R\right)=R\mu \left(1‐R/K\right)$ where μ is the maximum population growth rate of the prey and K represents prey carrying capacity or set to constant input $g\left(R\right)=I‐vR$, where I is the influx and v is the efflux rate. Scavenging rates are defined for the predator and scavenger to be

(10) $$q_{p}=\frac{s_{P}C}{1+f_{P}Rh_{\mathrm{RP}}+s_{P}Ch_{\mathrm{CP}}},$$

(11) $$q_{s}=\frac{s_{s}C}{1+f_{s}Rh_{\mathrm{RS}}+s_{s}Ch_{\mathrm{CS}}}.$$

### 3.2 Interactions at carcasses

We add an interference term to the model so that presence and density of the scavenger affects the handling time of carrion by the predator (Allen et al., 2014; Elbroch & Wittmer, 2013; Kane et al., 2017; Tallian et al., 2017). We use the parameter $n_{P}$ to determine the direction and magnitude of the effect of the scavenger on predator handling time. Handling time of the predator on the carrion takes the form

(12) $$h_{\mathrm{CP}}\left(S\right)=h_{CP0}+\frac{n_{P}y_{P}S}{1+y_{P}S}$$

where $h_{CP0}$ is the handling time for the predator in isolation, S is density of the scavenger, and $y_{P}$ is a scaling parameter for how much the density of the scavenger affects handling time of the predator. Handling time of the predator can be positively $\left(n_{P}>0\right)$ associated with scavenger density, as has been observed for example in brown bears scavenging wolf kills (Tallian et al., 2017), or negatively $\left(n_{P}<0\right)$ associated with scavenger density, as has been observed for example in bears (Krofel et al., 2012) or wolverines scavenging lynx kills (Mattisson, Andrén, et al., 2011). That the association between scavenger density and handling time can be positive or negative has been proposed to be the result of direct antagonistic interactions between predator species that take place near a carcass.

Similarly, the predator may also affect the scavenger. We model this through the parameter $n_{S}$ so the handling time of the scavenger on the carrion takes the form

(13) $$h_{\mathrm{CS}}\left(P\right)=h_{CS0}+\frac{n_{S}y_{S}P}{1+y_{S}P}$$

where $h_{CS0}$ is the handling time for the scavenger in isolation, P is density of the predator, and $y_{S}$ is a scaling parameter for how much the density of the predator affects handling time of the scavenger. However, we usually neglect interference by the predator on the scavenger by setting $n_{S}=0$. This is for simplicity and to focus on the primary predator. See Appendix Section A.5 for when $n_{S}\neq 0$ and Discussion for biological implications regarding changes in this parameter.

Other types of interactions between predator–scavenger pairs exist, for example, in lions and hyenas, there can be aggression and mortality not immediately linked to a carrion item, and golden eagles have been reported to kill bear cubs (Sørensen et al., 2008). However, we focus on behavioral interference more directly linked to scavenging. Furthermore, our model, built on classical foraging theory, includes many of the effects on scavenging by separating them out but does not consider that they may interact. For example, the consumption of a predator is affected by a scavenger through its subsequent feeding (scavenging) of the carcass, but this does not act through the energy conversion efficiency $a_{P}$ nor the proportion of killed prey immediately consumed when the kill occurs $\phi_{P}$. Nevertheless, our model is built to match the body of empirical measurements from the literature, for example, there are measures of scavenger effects on handling time but not of scavenger effects on energy conversion a or amount immediately consumed ϕ.

We do not include an intraspecific interference term in the functional response like in Beddington (1975) and DeAngelis et al. (1975). In contrast to these previous studies, we have attractions to carrion. In our model, we can have more attacks due to higher predator density. These previous studies only considered that it can happen one way, where they can only interfere with one another and decrease attacks. Furthermore, handling time (and the impact of the scavenger on the predator handling time) is included in scavenging propensity and the functional response, and we include a detailed discussion about why we expect this to be the case as well as what happens if it is not the case (see Section 3). In scavenging interactions, the outcome of the interference on handling time can be positive or negative (Krofel et al., 2012; Mattisson, Andrén, et al., 2011; Tallian et al., 2017) and we show how interference on handling time enters into the scavenging behavior functions and impacts predation.

### 3.3 Simplified models and further assumptions

We reduce the full model into two simplified models (Appendix SectionA.4.2, A.4.3 AA.4.2, A.4.3) representing limiting cases that allow us to better approximate that observed in nature and generate some analytical results, giving three models in total:

- Full dynamics model‐ using Equations (5), (6), (7), (8),
- R and C dynamics model, which allows only resource R and carrion C dynamics‐ using Equations 5 and 6 and setting the predator and scavenger populations to constant values, and
- No dynamics model, which is similar to Andren2011 where resource R, carrion C, scavengers S, and predators P are set to constant values, and kill rates and total kills are calculated using Equations 2, 3, and 4.

​

We use the three models for three following primary reasons: (a) in order to relate to and build upon previous work that considered no dynamics, (b) to represent reality where we know the systems have differences and that these differences need to be explained, and (c) to more fully explore parameter space. We make further simplifying assumptions to focus on the impact of the scavenger on the predator.

We assume the carrion pool, C, is generated by the predator with the proportion of prey biomass left as carrion given by $1‐\phi_{P}$. For realism and simplicity, we first assume the scavenger leaves no carrion, $\phi_{S}=1$, assuming this to be inaccessible to the main predator, for example, due to caching behavior (Mattisson et al., 2016). Alternatively, scavenger‐generated carrion may be accessible but not used by the main predator, as is often observed, for example smaller solitary felids rarely scavenging ursid kills (Krofel et al., 2012). This generates some asymmetry between the predator and scavenger, as both the predator and scavenger can feed from the carrion pool generated by the predator. For the more rare situation of closer symmetry in creating and accessing the carrion pool, for example hyena/lions (Amorós et al., 2020), see Appendix Section A.5 where we consider the carrion pool generated also by the scavenger killing prey, with $\phi_{S}<1$.

We assume the amount of prey 1‐ϕ remaining after immediate consumption is completely converted to carrion C and is then available for scavenging by the same or other species. If some amount of carrion biomass is lost, our model would assume that to occur through the parameter for carrion losses, δ. We generally consider the carrion pool to be available until it is completely scavenged, setting δ=0 so that there is no loss or decay due to other scavengers, decomposition, or the environment. Loss of carrion (due to other scavengers, decomposition, or the environment) lowers the steady state levels of scavengers, but it does not change results qualitatively (see Appendix Section A.6 for when δ>0). While the size of prey, who visited previously, and potentially decomposition status are known to affect scavenging of carrion, we do not consider their roles here. The roles of these factors may be considered in future theoretical work, although this will be challenging because it complicates the modeling and would likely require partial differential equation or individual‐based models to properly address their roles.

In order to reduce the model to focus on the key parameters that affect our question, we generally assume a constant near perfect energy conversion efficiency of biomass consumed being converted to biomass of the consumers so that $a_{P}=a_{S}=1$. When we analyze the Full dynamics models with nonperfect energy conversion efficiency, we do not see a qualitative change in our patterns (for an example on loss processes for when energy conversion parameters $a_{P}$ and $a_{S}$ are both <1 see Appendix Section A.6). Note that these conversion parameters $a_{P}$ and $a_{S}$ are absent from the two simpler models. We generally assume a minimum nonzero value for predation propensity f so that prey are consumed in the system, which allows the predators/scavengers to exist (otherwise they they would deplete all carrion and go extinct) in the Full dynamics model. Note that kill rates k are dependent on R even if predation propensity f is not related scavenging propensity s (we tested both by considering the model with and without a tradeoff in f and s).

We ran numerical simulations of each model using NDSolve in Mathematica v11 (Wolfram Research, Inc.). Initial conditions for each state variable were set to values away from the equilibrium, and simulations were allowed to run until no further change was observed in the state variables. We have found our results to be independent of these initial conditions and keep our attention to the positive equilibrium we obtained. We focus on this locally stable equilibrium (Appendix Section A.1) that we always found, as did Edwards (2001) in a somewhat dynamically similar model. Although we do not try to assess global stability, our simulations arrive at this equilibrium from initial values that are far from it (several orders of magnitude above or below the equilibrium value). We then assessed the resulting predation rates and pool sizes from these equilibrium conditions. We conducted a local stability analysis to show that this equilibrium is stable by looking at the sign (all negative) of the eigenvalues of the Jacobian matrix evaluated at the equilibrium (Appendix Section A.1).

We employ multiple types of theoretical analyses to create general results that do not rely heavily on particular parameters: (a) we use analytical analyses where possible, (b) we analyze parts or ingredients of the models to give more insight into how they behave, and (c) we use three different models arrayed along a gradient of dynamical feedbacks to cover more of modeling/parameter space. For the Full model and the two simpler models, we focused on parameter values that lead to positive values for densities of both the scavenger and predator in order to compare across models (see Table 1). The potential for coexistence of predators and scavengers and species dominance depends on the parameters in the Full model (Appendix Section A.2). We find coexistence to be possible when one species specializes more on live prey and the other species specializes more on carrion, thus has a higher scavenging propensity over some carrion densities (Figure 1, Appendix Section A.2).

Within that parameter value range that allows coexistence in the Full model and over a larger range (Table 1) in the two simpler models, we systematically varied parameters to assess predation along a gradient of scavenger density S. We focused on the role of scavenger mortality rate $m_{S}$ in the Full dynamics model as it determines scavenger density S and the coexistence region of parameter space. In the two simpler models, we directly manipulate scavenger density S, thus allowing us to answer our central question of how scavenger presence and abundance affects predation across all three models. We present general results that we found to be robust to our assumptions, not qualitatively sensitive to particular parameter values, and are supported by analytical results for the simpler model (Appendix Section A.4.3, and see Appendix Section A.7 for under what conditions our assumptions hold). We found that parameter value combinations in the ranges listed in Table 1 led to the general patterns depicted in the figures in the main text and Appendix A. The parameter values for those figures are listed in the figure legends. Where possible, we used foraging measurements reported in the literature to derive and further delimit parameter values (Appendix Section A.3.1).

To match our modeling output with empirical data, we did the following with eight empirical cases of predator–scavenger pairs: used the observed net effect and observed mechanisms to place it in model parameter and outcome space (one case), used the observed net effect and hypothesized mechanisms to place it in model parameter and outcome space (two cases), and predicted the net effect based on hypothesized mechanisms to place it in model parameter and outcome space (five cases).

## 4 RESULTS

### 4.1 Scavenger effects on abundance of predators, prey, and carrion

**FIGURE 3:**
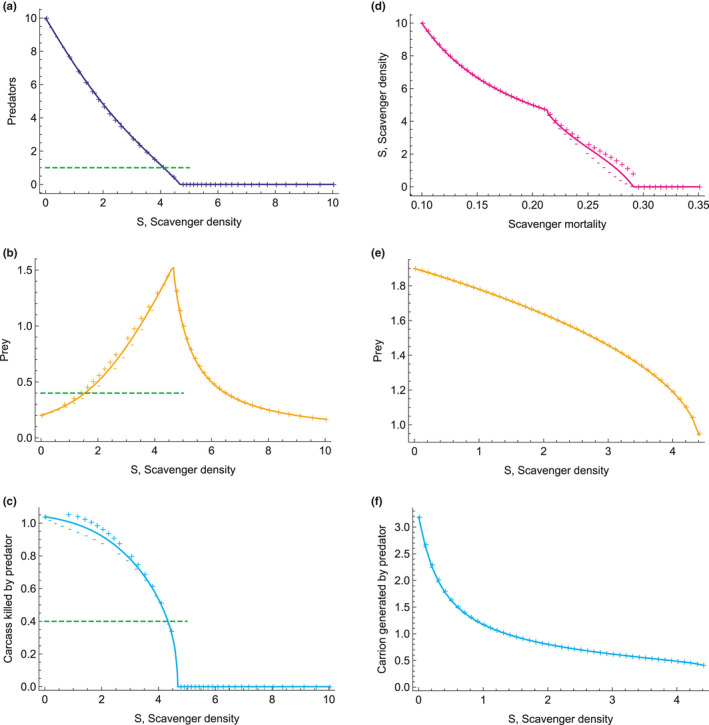
Effect of scavenger density S in the Full dynamics model for (a) predators P, (b) prey R, and (c) carrion C, where (d) scavenger density S is manipulated by changing scavenger mortality $m_{S}$. Scavenger density S is manually set in the R and C dynamics model. Effect of scavenger density S in the R and C dynamics model for (e) prey R, and (f) carrion C. Solid line is for when interference competition coefficient of scavenger on predator $n_{P}=0$, + points are for interference competition coefficient $n_{P}=1$ (increase in handling time of predator with scavenger) and — are for interference competition coefficient $n_{P}=‐1$ (decrease in handling time of predator with scavenger). Dashed green lines show the densities of (a) predators assumed in the R and C dynamics and No dynamics models, (b) prey assumed in the No dynamics model, and (c) carrion assumed in the No dynamics model for comparison. Parameter values are with accompanying figures for each model in Appendix SectionsA.4.1, A.4.2 AA.4.1, A.4.2

In the Full dynamics model, we manipulated scavenger mortality rate $m_{S}$, which directly affects the equilibrium scavenger density S (Figure 3d). Increasing scavenger abundance (decreasing scavenger mortality rate $m_{S}$) decreases predator abundance until predators go extinct (Figure 3a). This is because in the model with full dynamics, there is strong exploitative competition between the predators and scavengers for the prey and carcasses. The predators and scavengers have different mortality rates m, which affects their equilibrium densities (Equations A3 and A4), and thus, the total density of predators and scavengers declines as the density of scavengers increases until $m_{S}=m_{P}$ as depicted in Figure 3a when *S* = 10. Increasing scavenger abundance increases prey abundance until the predators go extinct (Figure 3b). Further increases in scavengers then decreases prey abundance, because then they are the main predator. Increasing scavenger abundance decreases carcass abundance until the predators go extinct and carcasses abundance reaches zero (Figure 3c). Further increases in scavengers has no effect on carcass abundance, because this carcass pool is generated only from the primary predator of the prey with these parameters (this assumption is relaxed in Appendix Section A.5). For the model with only R and C dynamics, increasing scavenger abundance decreases prey abundance and carcass abundance (Figure 3d,e).

### 4.2 Scavenger effects on total predation in the food web

**FIGURE 4:**
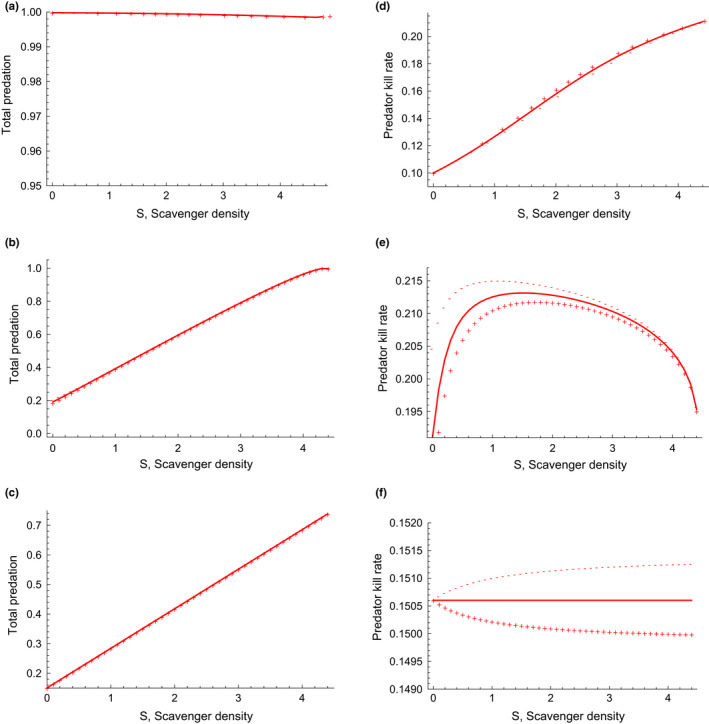
Change in total predation with increase in scavenger density when there is (a) Full dynamics, (b) only R and C dynamics, and (c) No dynamics. Change in predator kill rate with increase in scavenger density when there is (d) Full dynamics, (e) only R and C dynamics, and (f) No dynamics. Solid line is for when interference competition coefficient of scavenger on predator $n_{P}=0$, + points are for interference competition coefficient $n_{P}=1$ (increase in handling time of predator with scavenger) and — are for interference competition coefficient $n_{P}=‐1$ (decrease in handling time of predator with scavenger). Parameter values are with accompanying figures for each model in Appendix SectionsA.4.1, A.4.2, A.4.3

In the Full dynamics model, an increase in the scavenger population density has relatively little effect on total predation (Figure 4a). However, in the models with reduced dynamic feedbacks, that is, the model with R and C dynamics only (Figure 4b) or No dynamics model (Figure 4c, Table 2), total predation increases with an increase in scavenger density. The effect of interference competition by the scavenger on the predator, $n_{P}$, has a small effect on total predation for the Full dynamics model, R and C dynamics model (Figure 4b), and No dynamics model (Figure 4c). Relative to $n_{P}=0$, we see a decrease in total predation for $n_{P}>0$ (i.e., positive relation between handling time and scavenger density), and an increase in total predation for $n_{P}<0$ (i.e., negative relation between handling time and scavenger density).

**TABLE 2:** Effect of amount of dynamics (exploitative competition) allowed in the model on total predation and predator kill rate as scavenger density increases

	Full dynamics	R and C dynamics	No dynamics
Total predation	Slight decrease	Increases	Increases
Predator kill rate	Increases	Increases/decreases	Increases, 0, decreases

Note

The interference competition specified by $n_{P}$, the effect of scavenger on predator handling time, also affects predation metrics and is why the predator kill rate for the No dynamics model has increases, 0, decreases for $n_{P}=‐1$, $n_{P}=0$, $n_{P}=1$, respectively.

### 4.3 Scavenger effects on predator kill rate

**FIGURE 5:**
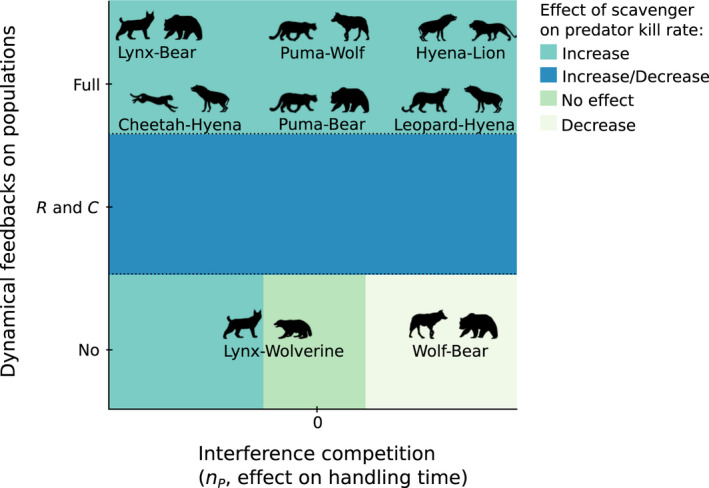
Effect of an increase in scavenger population density on predator kill rate as determined by interference competition (horizontal axis) and dynamical feedbacks on populations (exploitative competition) in the food web (vertical axis). Interference competition is defined as $n_{P}$, scavenger influence on predator handling time. An $n_{P}<0$ is a decrease in handling time of predator with scavenger, and an $n_{P}>0$ is an increase in handling time of predator with scavenger. Dynamical feedbacks on populations are distinguished by the three models: Full is Full dynamics model, R and C is resource and carrion dynamics model, and No is No dynamics model. Colored regions indicate the relationship of predator kill rate with scavenger density. Increase/Decrease denotes an increase followed by a decrease with scavenger density. Animal silhouettes (from phylopic.org) on top of the phase plot are empirical examples of predator–scavenger pairs taken from the literature. Placement of the coupled predators–scavengers is based on evidence from the literature, with some being predictions based on parameters and others being results based on hypothesized parameters. The “Lynx–Bear” comes from Krofel et al. (2012) and is an observed net effect with hypothesized mechanisms. The “Wolf–Bear” comes from Tallian et al. (2017) and is an observed net effect with hypothesized mechanisms. The “Lynx–Wolverine” comes from Mattisson, Andrén, et al. (2011) and is an observed net effect (usually 0, but can be +) with some mechanisms measured in López‐Bao et al. (2016). All other pairs are taken from Krofel et al. (2012) and original sources within and are predictions of the net effects, while the real net effects remain unknown. Note that all pairs are arrayed along the vertical axis based on the hypothesized natural density regulation, and this may vary across different management regimes

Predator kill rate, $k_{P}$ increases with increasing scavenger density S for the Full dynamics model (Figure 4d), whereas it increases and then decreases with S for the R and C dynamics model (Figure 4e) and is relatively constant with S for the No dynamics model (Figure 4f). Relative to when there is no interference competition by the scavenger on the predator, $n_{P}=0$, we see a small increase in predator kill rate for $n_{P}>0$ and a decrease in predator kill rate for $n_{P}<0$ in the Full dynamics model. This contrasts with a decrease in predator kill rate for $n_{P}>0$ and an increase in predator kill rate for $n_{P}<0$ in the models with reduced dynamics. The combined effects of the amount of dynamics and species interactions on predator kill rate can be seen in Table 2 and Figure 5. We see that how predator kill rate changes with scavenger density is strongly determined by the amount of dynamics (exploitative competition) allowed in the model. However, the effects of interference competition by the scavenger on the predator determines the sign of the effect in the No dynamics model. Some of the relationships of predator kill rates to scavenger density reflect the nonlinear changes in pool sizes of resources. In the Full dynamics model, pool sizes are changing with scavenger density but to different degrees (Figure 3). Predator kill rate increases in the Full model as both carrion declines and prey increases as predators are competitively replaced by scavengers (Figure 3). In the R and C dynamics model, as scavenger density increases, both R and C decline while P is constant (Figure 3). Total predation increases with scavenger density (Figure 4b). Predator kill rate goes up sharply initially because carrion C declines sharply as scavenger density increases in the system. Since scavenging strategy follows changes in carrion density closely, the predator must kill more because there is less carrion C. As scavenger density S further increases, resource R decreases too and kill rate decreases. Thus, the predation pattern is driven by depletion of carrion C and depletion of overall resources by scavengers S.

The effect of increasing scavenger density on the kill rate of the predator in the No dynamics model depends on $n_{P}$, the interference competition as manifested by the scavenger affecting the carrion handling time of the predator. Scavenger density S affects predator carrion handling time $h_{\mathrm{PC}}$ and scavenging strategy s in opposite ways, which makes it difficult to predict how kill rates are affected. However, we are able to show analytically that under certain assumptions, S decreases predator kill rate if it increases predator handling time (Appendix Section A.4.3). Analytical techniques are especially useful if $h_{\mathrm{PC}}$ is not in the scavenging strategy equation, s, that is, for a predator behaving non‐adaptively. This may be the case for wolves and bears but is unlikely to be true for lynx, wolverines, or cheetahs Hilborn2018. If $h_{\mathrm{PC}}$ is not in the scavenging strategy equation, s, increasing $h_{\mathrm{PC}}$ always decreases predator kill rate, so if $n_{P}>0$ and scavenger density S increases, predator kill rate will always decrease (Appendix Section A.4.3).

### 4.4 Comparison to empirical examples

The food‐web topology in our model resembles that observed in nature; thus, we are able to match our assumptions and predictions with many empirical examples. Most interspecific interactions are asymmetric, with one species more likely to gain access to and stay at a carcass, for example, lynx and wolverines (Mattisson, Andrén, et al., 2011) and in wolves (Tallian et al., 2017) or solitary cats (Hilborn et al., 2018) and ursids (Krofel et al., 2012). The interaction between lions and hyenas is perhaps the only approximately symmetrical interaction. In this case, the scavengers contribute significantly to the carrion pool and the predators affect scavenger handling times (see Appendix Section A.5).

The empirical examples of predator–scavenger pairs (Krofel et al., 2012) appear to be spread throughout the model parameter space when we overlay them on to the phase plot of how predator kill rate is affected by scavenger density (Figure 5). We placed the predator–scavenger pairs based on available evidence—predictions based on parameters derived from the literature and results in the literature allowing us to hypothesize the parameters. For example, in the lynx and wolverine interaction, lynx appears to quickly abandon a carcass when a wolverine is present, thus a negative $n_{P}$ effect of scavenger on handling time of the predator. This can decrease the time until their next kill, so there can be an increase in kill rate (aqua colored region) and exploitative competition can be high (López‐Bao et al., 2016). Thus, the “Lynx–Wolverine” is both a prediction based on parameters and an observed result of the interaction (Mattisson, Andrén, et al., 2011). In a lynx and bear system, bears found 32% of lynx‐killed prey and lynx lost 15% of their prey biomass to bears, which resulted in a 23% increased lynx kill rate; thus, “Lynx–Bear” in aqua colored region of Figure 5 is an observed net effect with hypothesized mechanisms. The increased kill rate, however, interestingly for this case, did not fully compensate for their losses to bears (Krofel et al., 2012).

In the wolf and brown bear interaction, the direct interference competition can be high, which may result in the observed decrease in kill rates (“Wolf–Bear” in white colored region of Figure 5) (Tallian et al., 2017). Bears appear to be dominant and able to displace wolves from a carcass; however, wolves may linger and increase time until their next kill, thus a positive $n_{P}$ effect of scavenger on handling time of the predator. This interaction between wolves and brown bears is important in Yellowstone to understand the whole ecosystem effects of the return of wolves there (Massey et al., 2013). The species identity in these predator–scavenger pairs often determines the interactions (Allen et al., 2015), for example, unlike brown (grizzly) bears, black bears often lose prey to wolves. The remaining predators–scavenger pairs in Figure 5 are taken from Krofel et al. (2012) and original sources therein. These are all predictions of the net effects considering also their hypothesized natural density regulation (management regime) that determines their placement in one of the three models arrayed along the vertical axis.

## 5 DISCUSSION

### 5.1 Key results

We built a dynamical model of scavenging based on foraging theory and expanded on it to include two facultative scavenger species, which we refer to as the predator and scavenger. Building on previous work, we provide nontrivial insights on ecological food webs showing that the effect of the interaction between predators and scavengers on equilibrium population sizes can vary depending on the context (Abrams, 1987). Generally, the addition of a scavenger to a food web has effects on the kill rate of the predator. The magnitude and sign of those effects depend on the architecture of the system, primarily determined by the management regime that affects the population dynamics of the predators and scavengers (Figure 5). Only under some circumstances should the abundance of scavengers have absolutely no effect on kill rates: when they result in both no changes in the predation strategy of the main predator and no changes in predator and prey densities, perhaps a highly unlikely scenario. Scavengers still increase total losses of prey in this case (Figure 4c).

When predator, scavenger, and prey abundances are kept constant by management (No dynamics model), scavengers have minimal effects on predator kill rate. This changes with the inclusion of dynamics in predators, scavengers, and/or prey. For example, the addition of scavengers forces the predators to kill more over the entire abundance range of scavengers in the Full dynamics model and over some abundance range of scavengers in the R and C dynamics model (Figures 4, 5). This should be a fundamental prediction—scavengers most likely increase predator kill rates, especially if there are feedback effects on the prey or carrion resources.

A system with both predators and scavengers can increase prey density as depicted in Figure 3b and described in the first paragraph of Section 3. Scavengers increase prey density because they decrease predator density through competition in the Full dynamics model. Specifically, even though scavengers increase prey density, predators also depend on carrion and prefer carrion to prey (just not as strongly as scavengers do). Thus, when scavengers come into the system, they decrease carrion density and even though they increase prey density, they still decrease predator density. This nonintuitive result highlights the importance of constructing a full dynamical model for this situation. This will happen in the full dynamical model if the invasion criteria is met for S at P equilibrium (see Appendix Section A.2). This effect of competition may be realistic in some systems but not in others, and it may be that we do not see it because predators are under so much anthropogenic control. In fact, if the predators are highly controlled and therefore effectively push the system to the R and C dynamics model, the effect disappears, in which case, a system with both predators and scavengers can also decrease prey density as scavenger density increases (Figure 3d). This system, as described by the R and C dynamics model, has not the same constraints on total predators and scavengers as described by the Full dynamics model. That is why it is important that we test the three different models and analyze their outcomes.

For the No dynamics model, the relationship of predator kill rate with scavenger density is flat if the effect of scavengers on predator handling time is negligible ($n_{P}=0$), with some small effect for $n_{P}>0$ or $n_{P}<0$ (Figure 4f). This means that most effects from the addition of scavengers on predator kill rates are through dynamical feedbacks. It is noteworthy that interference competition ($n_{P}$) has relatively more of an effect in the simple model with no dynamics than in the other models. This suggests that interference competition plays a larger role when the system dynamics are fully constrained by management.

### 5.2 Relationship to previous work, observations, and caveats

Andrén et al. (2011) defined kill rate as the number of reindeer killed per predator per time unit, but they kept total number of predators (lynx) and scavengers (wolverines) constant and just changed the ratio of predators to scavengers. They found that the expected number of reindeer killed per predator increases as there are more lynx (less wolverines) in the system. Our No dynamics model is somewhat comparable, because it lacks population feedbacks, while behavioral strategy feedbacks always exist in our models. We find relatively constant predator kill rates with increases in scavenger numbers, but we do not hold total number of predators and scavengers constant. Our Full dynamics model is also similar to the model of Andrén et al. (2011)—the total number of predators and scavengers combined is constrained by the total energy in the ecosystem. However, we find relatively constant total predation as we shift the ratio of predators (lynx) to scavengers (wolverines). Our models contribute to a more complete understanding of predation on reindeer by building on Andrén et al. (2011) to predict which factors are important and what the overall outcome is when multiple predator species live in proximity to one another and prey on this ecologically and economically important species (Mattisson, Odden, et al., 2011; Pedersen et al., 1999; Tab lado et al., 2014).

Large carnivores acting as predators have major impacts on ecosystems (Ripple et al., 2014) but scavengers, represented by many of the same and different species spread across all biomes (Moleón & Sánchez‐Zapata, 2015), may have similar impacts. Other types of scavengers should be examined as well since they can have an even bigger role than carnivorous mammals in some systems (Henden et al., 2014). An ecosystem may be able to support more obligate‐type scavengers if the primary predator/scavenger does not use all the carrion. If these additional scavengers remove carrion that the primary predator/scavenger would intend to use, then we predict that this can also increase kill rates of the predator (Table 2). Vultures may be one of only a few obligate scavengers in terrestrial systems, but they consume a small or negligible portion of biomass compared with lions and hyenas, and large carnivores in general are able to defend their kills from vultures (Moleón et al., 2014). The savannah system has a number of exemplary predator/scavenger species including vultures, hyenas, lions, jackals, and many herbivores like zebra, springbok, wildebeest, oryx, and elephants (Getz, 2011). Vultures, lions, and hyenas generally consume 100% of medium and large carcasses in this system (Moleón & Sánchez‐Zapata, 2015). Thus, scavenging and the interactions between predator species play a large role in biomass transformation rates in many different ecosystems.

We focus our modeling analysis primarily on how predation is affected by scavenging in a simplified food web, assuming one of the main mechanisms is the effect of scavengers on predators through exploitative resource competition and direct interference. For simplification and realism, we generally assume a mostly one‐sided interaction of the scavenger affecting the predator and not the predator impacting the scavenger much except through prey depletion and carrion generation. We relax these assumptions and consider a symmetrical interaction between predators and scavengers in creating and accessing the carrion pool, and in interference competition (Appendix Section A.5), to show this does not have a big impact on our results. However, our model does ignore some of the other complexity observed in food webs. For example, although predators provide huge amounts of carrion to scavengers, they can increase scavenger mortality (Prugh & Sivy, 2020). Manipulating the carrion pool through carcass provisioning for some scavenger species may also attract or increase predation by scavengers on other prey species and change prey abundances and spatial distributions (Cortés‐Avizanda et al., 2009; Cortés‐Avizanda et al., 2009; Fielding et al., 2014).

### 5.3 Management implications

For wildlife management purposes, we would reiterate that the importance of the predator/scavenger interaction depends on the goal of the management. Pool sizes of predators, scavengers, or prey may all be the target of management. Note, however, that many predator/scavenger species such as insects and marine invertebrates are not managed, while some facultative scavenging birds are actively managed to promote their endangered populations (Cortés‐Avizanda, Carrete, et al., 2009; Margalida et al., 2012). In addition, the ratio of predators to scavengers has been suggested as a potential management target (Andrén et al., 2011; Mattisson, Andrén, et al., 2011). Management may also be targeted towards ecosystem processes or rates, such as kill rates. Here, we show that while often discussed and measured, these rates are complex aggregate measures of many interacting underlying ecological processes that vary with context. This may make it difficult to understand changes in ecosystem structure based strictly on these rates.

Management that controls predator and scavenger populations to keep them at low numbers as well as regulate prey and carrion abundance prevents many natural ecosystem feedbacks. Such actions reduce the probability that predators increase their kill rate when their prey carrion is eaten by scavengers. This is also where human harvesting of the same prey population can have some influence on the dynamics. However, for more natural systems or in situations where management is not controlling predator and prey numbers, feedbacks make it likely that predators may increase kill rates when their prey carrion is eaten by scavengers. This can occur even if only prey and carrion abundance is allowed to respond dynamically due to consumption, as seen in the R and C dynamics model (Figure 4e).

Managers of prey populations likely care most about total losses and need to know whether expected kill rates from predators/scavengers should be tallied independently of one another to get the total losses in the prey population in an area. We show that, in many cases, losses from predator/scavengers are additive, for example, lynx and wolverines together are likely additive. However, in other cases or where feedbacks occur, total losses are relatively constant (Figure 4a), so the predators compensate for one another. In some cases, for example wolves and bears together, although wolf (predator) kill rates may go down, total losses may still increase (Table 2), a previously unknown insight (Tallian et al., 2017). The question that follows is whether managers should try to manage the predators/scavengers in a way that keeps them spatially separated. We suggest that it depends on the predators and if management will allow the populations to grow to their full potential. The general result is, however, given by the total energy constraint on the ecosystem, more predators/scavengers are not supported by keeping them together (Figure 3).

### 5.4 Future work

We provide a roadmap to outcomes (Figure 5). Our models are able to reproduce the different patterns observed in predator/scavenger pairs in nature. This ability provides some support to our model, meaning we may use it to explain the cases and differences between the predator–scavenger pairs. Future empirical studies could be designed to evaluate these predictions. Based on our analysis, data collection efforts should be focused on quantifying the scavenger effect on handling time of the predator, and the amount of population feedbacks in the systems, which perhaps can be extracted from the numerical response. These are the key variables that distinguish the systems and allow us to predict whether scavenging will increase or decrease kill rates. One of the fundamental differences we use to distinguish the systems is how the species interaction affects handling times. However, we note that interference competition may interact with exploitative competition through the carrion pool size. This suggests that exploitative competition needs to be evaluated; thus, carcass density is important and should be measured. Furthermore, if the parameters that change the magnitude and shape of the scavenging response to carrion density, z and b, were to be measured on different scavengers, they would provide key information.

## 6 CONCLUSION

Scavenging can impact predation through multiple direct and indirect pathways: by changing the kill rates of predators, by decreasing available carrion, by bringing predators/scavengers into more direct contact and causing interference, and by changing growth rates of predator/scavenger populations. The importance of these pathways will vary between food webs depending on the identity of the predator/scavenger pairs, which determines their interactions, and how the populations of predators/scavengers are controlled. We suggest this is the reason for the different, and sometimes opposite effects seen, of the presence of scavengers on predator kill rates. Our hope is that this modeling provides a useful framework for predicting and understanding the effect of scavenging on predation across food webs in different types of ecosystems.

## CONFLICT OF INTEREST

The authors declare no conflict of interests.

## AUTHOR CONTRIBUTIONS

**Jarad P. Mellard:** Conceptualization (lead); Formal analysis (lead); Investigation (lead); Methodology (lead); Project administration (lead); Writing—original draft (lead); Writing—review and editing (lead). **Sandra Hamel:** Conceptualization (supporting); Resources (supporting); Writing—review and editing (supporting). **John‐André Henden:** Conceptualization (supporting); Resources (supporting); Writing—review and editing (supporting). **Rolf A. Ims:** Conceptualization (supporting); Funding acquisition (lead); Resources (supporting); Writing—review and editing (supporting). **Audun Stien:** Conceptualization (supporting); Formal analysis (supporting); Investigation (supporting); Methodology (supporting); Resources (supporting); Writing—review and editing (supporting). **Nigel Yoccoz:** Conceptualization (supporting); Resources (supporting); Writing—review and editing (supporting).

## Figures and Tables

**FIGURE A1:**
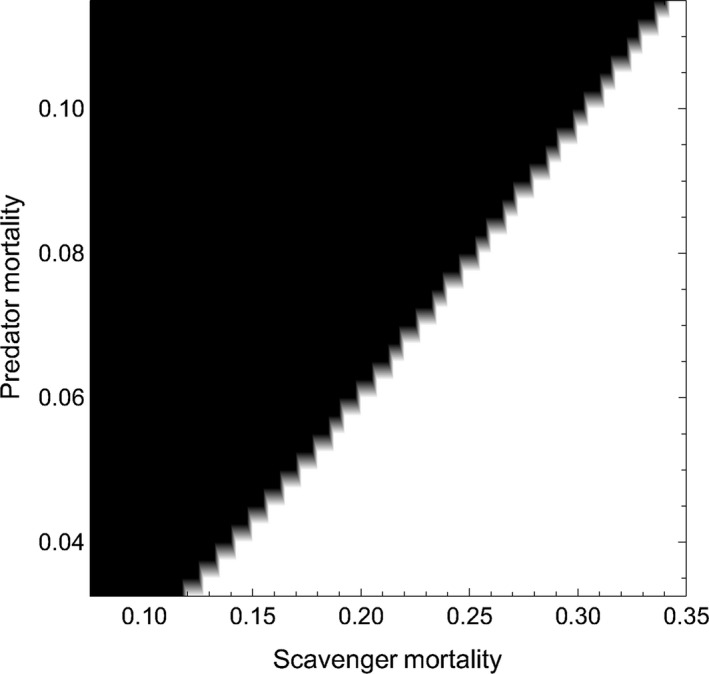
Scavenger can invade $\frac{\mathrm{dS}}{\mathrm{Sdt}}>0$ the system (in shaded region) with the predator present. Parameter values are $\phi_{P}=0.465,\phi_{S}=1,m_{P}=0.1,h_{\mathrm{RP}}=4,h_{\mathrm{RS}}=4,h_{CP0}=1,h_{\mathrm{CS}}=1,b_{P}=1,b_{S}=1,z_{P}=0.1,z_{S}=0.7,a_{P}=1,a_{S}=1,I=1,v=0.001,\delta =0$

**FIGURE A2:**
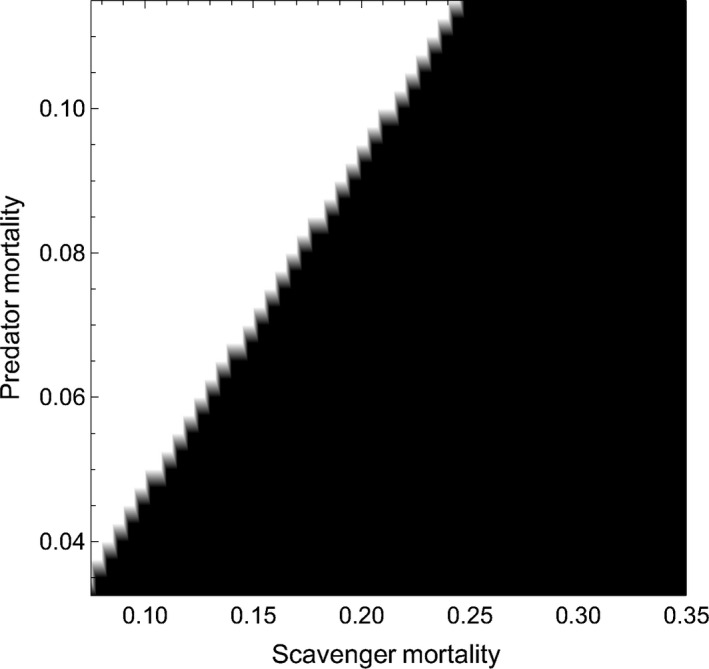
Predator can invade $\frac{\mathrm{dP}}{\mathrm{Pdt}}>0$ the system (in shaded region) with the scavenger present. Parameter values are $\phi_{P}=0.465,\phi_{S}=1,m_{P}=0.1,h_{\mathrm{RP}}=4,h_{\mathrm{RS}}=4,h_{CP0}=1,h_{\mathrm{CS}}=1,b_{P}=1,b_{S}=1,z_{P}=0.1,z_{S}=0.7,a_{P}=1,a_{S}=1,I=1,v=0.001,\delta =0$

**FIGURE A3:**
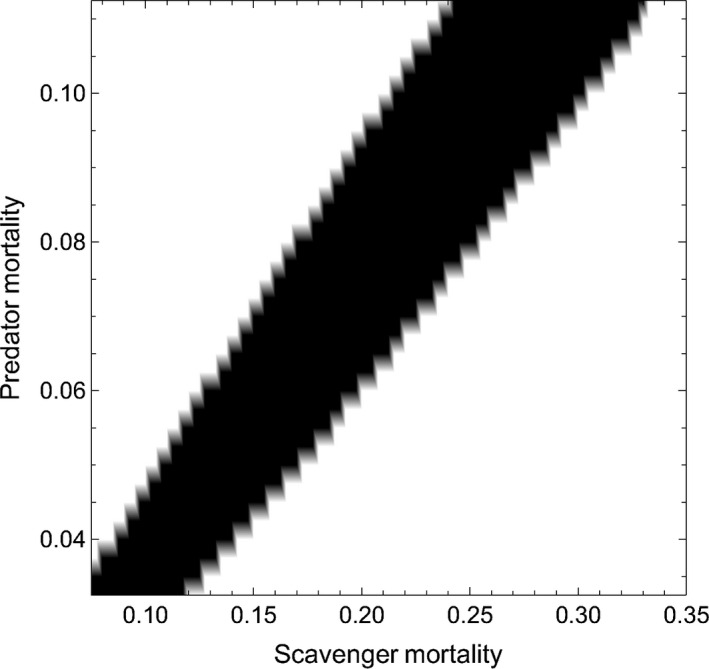
Example parameter combinations for coexistence in shaded region. Parameter values are $\phi_{P}=0.465,\phi_{S}=1,m_{P}=0.1,h_{\mathrm{RP}}=4,h_{\mathrm{RS}}=4,h_{CP0}=1,h_{\mathrm{CS}}=1,b_{P}=1,b_{S}=1,z_{P}=0.1,z_{S}=0.7,a_{P}=1,a_{S}=1,I=1,v=0.001,\delta =0$

**FIGURE A4:**
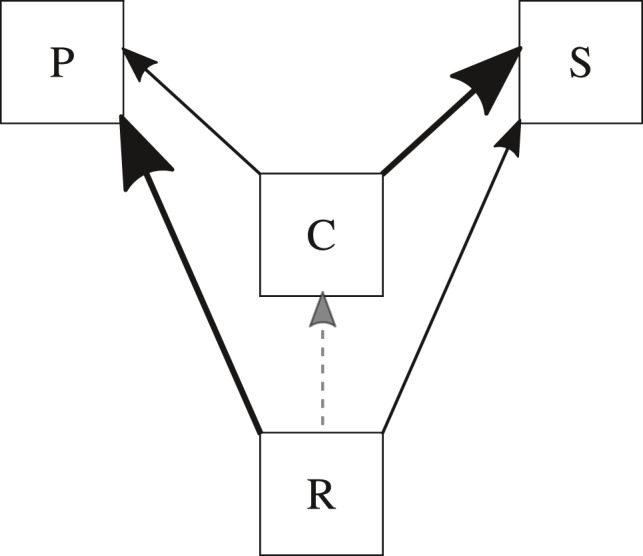
Full model with state variables for prey resource R, carrion C of prey killed by primary predators P and scavengers S, both of whom are facultative scavengers. Lines connecting state variable boxes represent potential energetic (biomass) pathways, with thickness of solid lines and size of arrows indicating relative specialization of the predator and scavenger on the two types of resources R and C, where dashed line indicates conversion of prey resource R to carrion C

**FIGURE A5:**
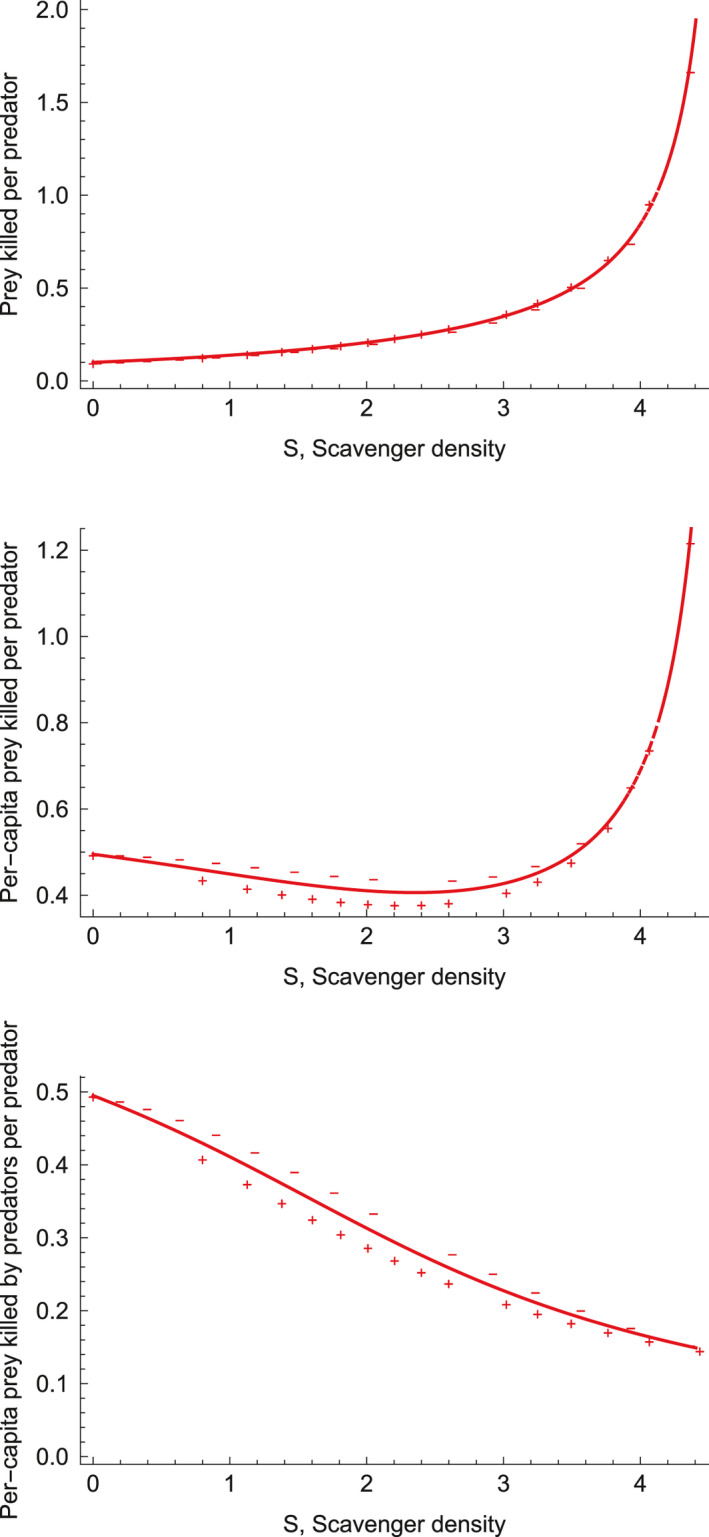
Predation metrics affected by scavenger density and defined as Prey killed per predator is total predation per predator, Per‐capita prey killed per predator is total predation per prey per predator, and Per‐capita prey killed by predators per predator is predator kill rate per prey as mathematically defined previously in Appendix Section A.3.2. Solid line is for when interference competition coefficient of scavenger on predator $n_{P}=0$, + points are for interference competition coefficient $n_{P}=1$ (increase in handling time of predator with scavenger) and — are for interference competition coefficient $n_{P}=‐1$ (decrease in handling time of predator with scavenger). Parameter values for figures of this model are $\phi_{P}=0.465,\phi_{S}=1,m_{P}=0.1,h_{\mathrm{RP}}=4,h_{\mathrm{RS}}=4,h_{CP0}=1,h_{\mathrm{CS}}=1,b_{P}=1,b_{S}=1,z_{P}=0.1,z_{S}=0.7,a_{P}=1,a_{S}=1,I=1,v=0.001,\delta =0$ (see Table 1 for definitions)

**FIGURE A6:**
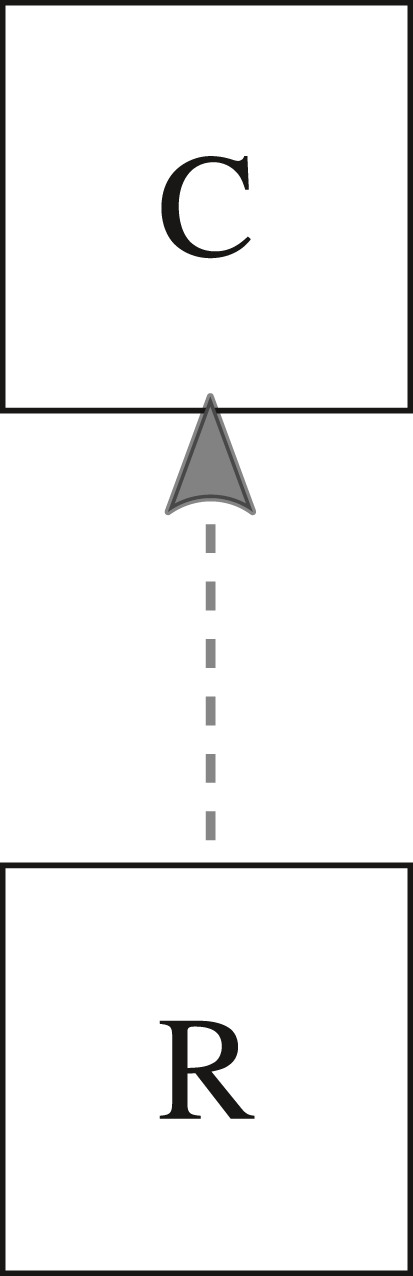
R and C dynamics model with state variables prey resource R and carrion killed by predator,C where dashed line indicates conversion of resource R to carrion C

**FIGURE A7:**
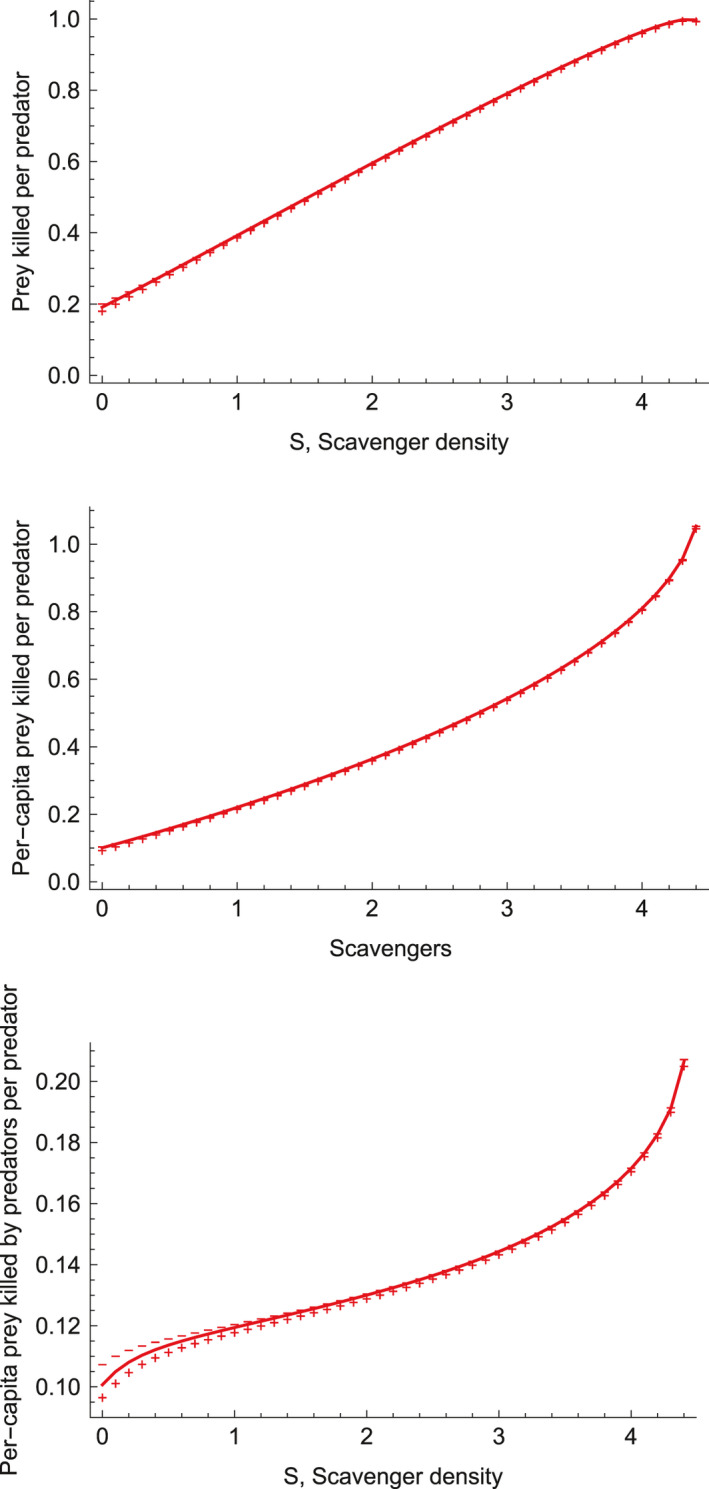
Predation metrics affected by scavenger density and defined as Prey killed per predator is total predation per predator, Per‐capita prey killed per predator is total predation per prey per predator, and Per‐capita prey killed by predators per predator is predator kill rate per prey as mathematically defined previously in Appendix Section A.3.2. Solid line is for when interference competition coefficient of scavenger on predator $n_{P}=0$, + points are for interference competition coefficient $n_{P}=1$ (increase in handling time of predator with scavenger) and— are for interference competition coefficient $n_{P}=‐1$ (decrease in handling time of predator with scavenger). Parameter values for figures of this model are $P=1,K=2,\mu =2,m_{P}=0.1,h_{\mathrm{RP}}=4,h_{\mathrm{RS}}=4,h_{CP0}=1,h_{\mathrm{CS}}=1,b_{P}=1,b_{S}=1,z_{P}=0.1,z_{S}=0.7$ (see Table 1 for definitions)

**FIGURE A8:**
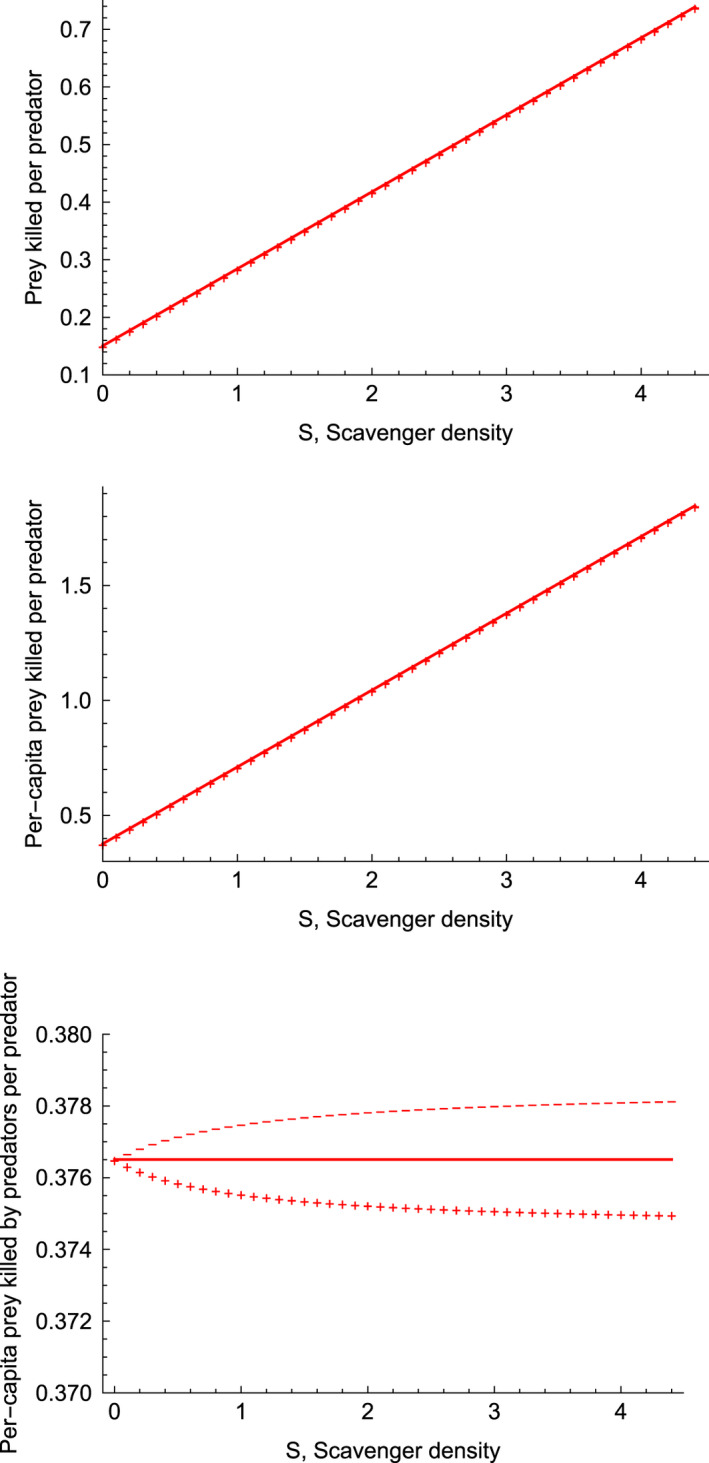
Predation metrics affected by scavenger density and defined as Prey killed per predator is total predation per predator, Per‐capita prey killed per predator is total predation per prey per predator, and Per‐capita prey killed by predators per predator is predator kill rate per prey as mathematically defined previously in Appendix Section A.3.2. Solid line is for when interference competition coefficient of scavenger on predator $n_{P}=0$, + points are for interference competition coefficient $n_{P}=1$ (increase in handling time of predator with scavenger) and‐ are for interference competition coefficient $n_{P}=‐1$ (decrease in handling time of predator with scavenger). Parameter values for figures of this model are $P=1,C=0.4,R=0.4,h_{\mathrm{RP}}=4,h_{\mathrm{RS}}=4,h_{CP0}=1,h_{\mathrm{CS}}=1,b_{P}=1,b_{S}=1,z_{P}=0.1,z_{S}=0.7$ (see Table 1 for definitions)

**FIGURE A9:**
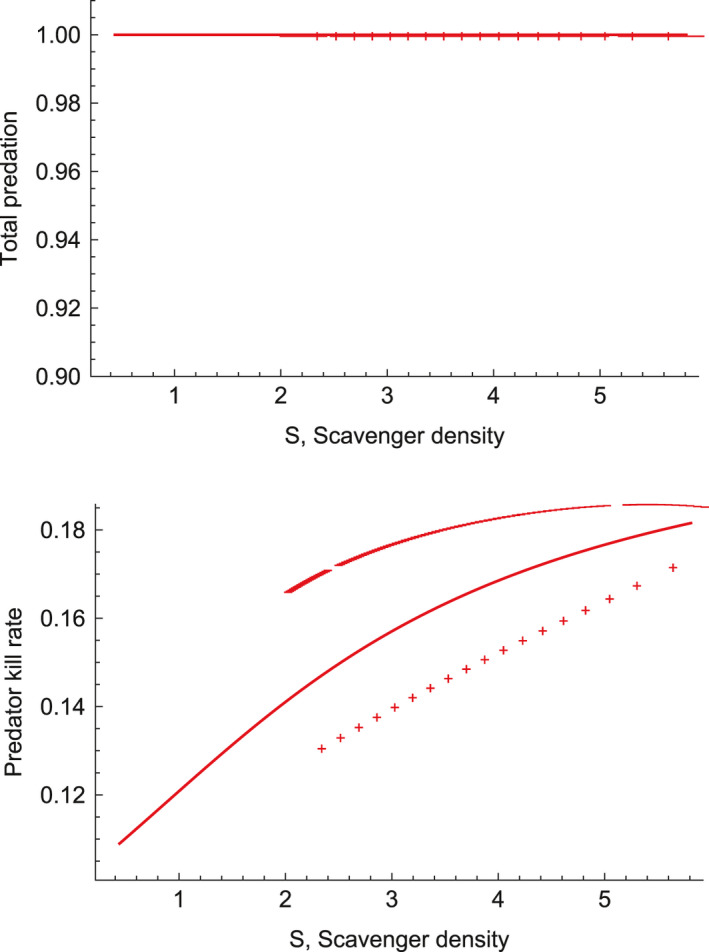
Change in total predation and predator kill rate with increase in scavenger density. Solid line is for when interference competition coefficient of scavenger on predator $n_{P}=0$, + points are for interference competition coefficient $n_{P}=1$ (increase in handling time of predator with scavenger) and — are for interference competition coefficient $n_{P}=‐1$ (decrease in handling time of predator with scavenger). Parameter values for figures of this model are $\phi_{P}=\phi_{S}=0.465,m_{P}=m_{S}=0.1,h_{\mathrm{RP}}=4,h_{\mathrm{RS}}=4,h_{CP0}=1,h_{\mathrm{CS}}=1,b_{P}=1,b_{S}=1,z_{P}=0.1,z_{S}=0.7,a_{P}=1,a_{S}=1,I=1,v=0.01$ (see Table 1 for definitions)

**FIGURE A10:**
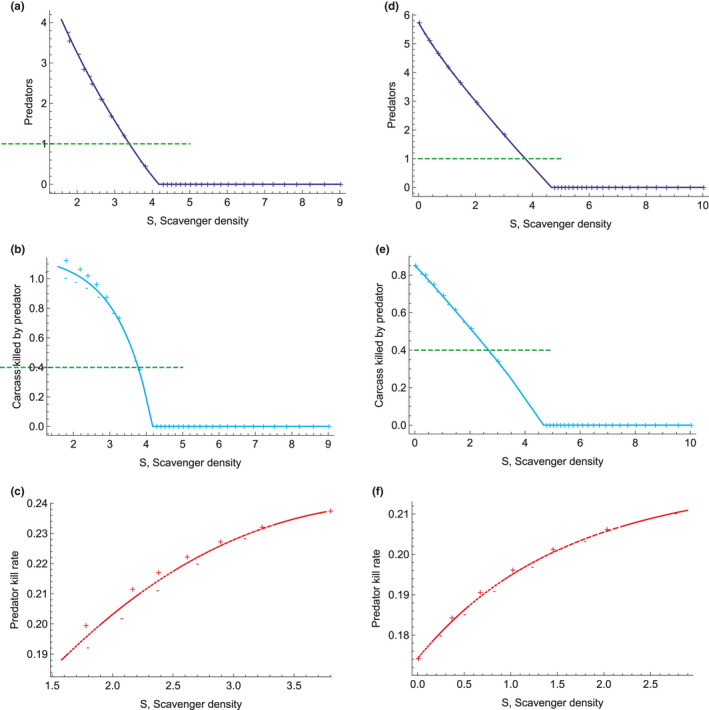
Effect of scavenger density S in the Full dynamics model for (a,d) predators P, (b,e) carrion C, and c,f) predator kill rate for different parameter values, (a,b,c) energy conversion to predators and scavengers $a_{P}=a_{S}=0.9$ and δ=0.1 and (d,e,f) energy conversion to predators and scavengers $a_{P}=a_{S}=1$ and δ=0.5. Scavenger density S is manipulated by changing scavenger mortality $m_{S}$. Solid line is for when interference competition coefficient of scavenger on predator $n_{P}=0$, + points are for interference competition coefficient $n_{P}=1$ (increase in handling time of predator with scavenger) and ‐ are for interference competition coefficient $n_{P}=‐1$ (decrease in handling time of predator with scavenger). Dashed green lines show the densities of (a) predators assumed in the R and C dynamics and No dynamics models, (b) carrion assumed in the No dynamics model for comparison. Parameter values for figures of this model are $\phi_{P}=0.465,\phi_{S}=1,m_{P}=0.1,h_{\mathrm{RP}}=4,h_{\mathrm{RS}}=4,h_{CP0}=1,h_{\mathrm{CS}}=1,b_{P}=1,b_{S}=1,z_{P}=0.1,z_{S}=0.7,I=1,v=0.001$ (see Table 1 for definitions)

**FIGURE A11:**
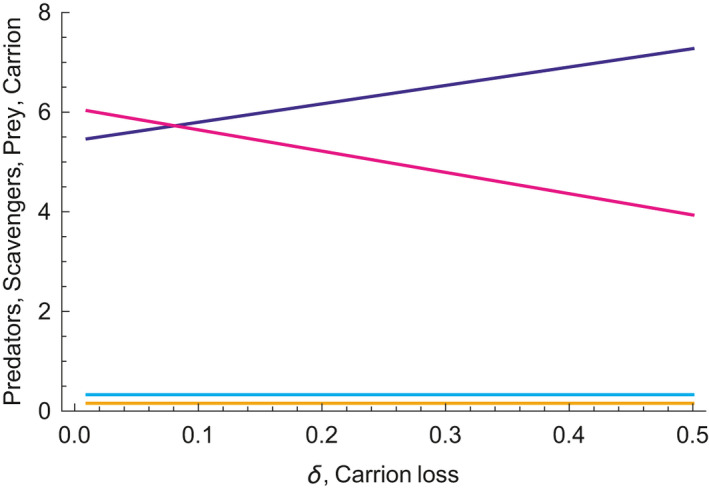
Effect of carrion loss, δ on equilibrium predator P density (blue), scavenger S density (magenta), prey R density (orange), and carrion C density (aqua). Parameter values for figures of this model are $\phi_{P}=0.465,\phi_{S}=1,m_{P}=0.1,m_{S}=0.12,h_{\mathrm{RP}}=4,h_{\mathrm{RS}}=4,h_{CP0}=1,h_{\mathrm{CS}}=1,b_{P}=1,b_{S}=1,z_{P}=0.1,z_{S}=0.7,I=1,v=0.001$ (see Table 1 for definitions)

**FIGURE A12:**
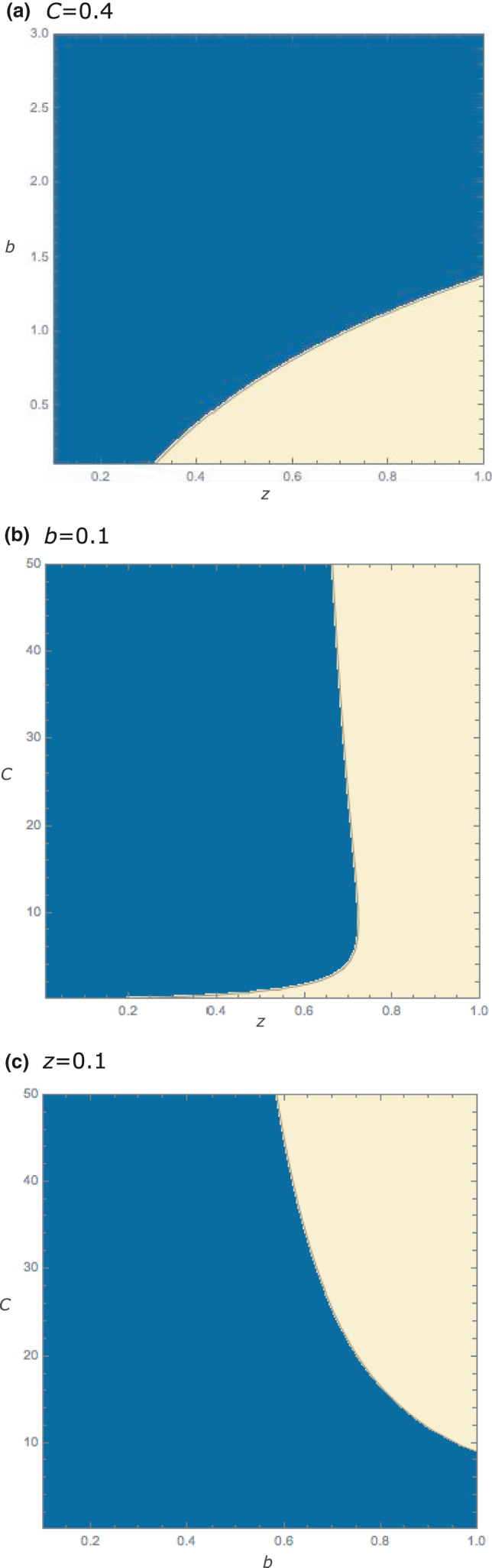
Parameter space where predator kill rate decreases with handling time (blue region), specifically the derivative of the predator kill rate <0. Parameter values for these figures are (a) C=0.4, (b) b=0.1, and (c) z=0.1, with other parameters $P=1,R=0.98,h_{\mathrm{RP}}=4,h_{\mathrm{CP}}=1$ (see Table 1 for definitions)

**FIGURE A13:**
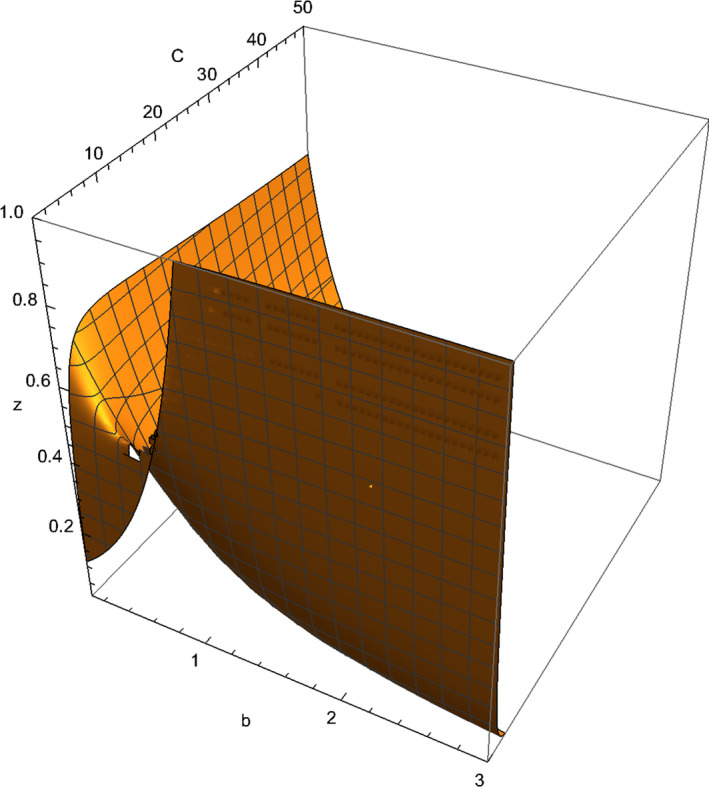
Parameter space where predator kill rate decreases with handling time, specifically the derivative of the predator kill rate <0 separated by the 3‐dimensional contour. Parameter values for this figure correspond to previous Figure A12 that takes 2‐dimensional slices through parameter space

## Data Availability

No original data were collected for or used in this study.

## APPENDIX A

### A.1 Equilibrium analysis

The formulation for the equilibrium solution for Equations 5–(9) is difficult to read; thus, we simplify the equations in order to show the algebraic formulation here, which is still fairly complicated but is better than no analytical results. We used this slightly simplified model to generate some analytical results for the equilibria and note that it can be used as an approximation of the Full model that we use in the numerical simulations. In the simplified model, we assume simpler Type I forms for kill rates and scavenging rates, $k_{i}\left(R\right)=k_{i}R$ and $q_{i}\left(C\right)=q_{i}C$, which assume linear responses for predators and scavengers on the food sources. In the Full model, we employ a Type II form and use that in the main text for realism, to match prior theoretical work with the disk equation, and to bring new insights into effects of scavenging within that framework. At equilibrium, we have not found major differences in the output of these two forms. In the simplified model, we also assume that $g\left(R\right)=I‐vR$, and we also know that $g\left(R\right)=I$ leads to a very similar form and the same conclusions. The only condition we know of that our simplified model does not address well is if we assume $g\left(R\right)=R\mu \left(1‐R/K\right)$, because this can lead to an unstable region of parameter space, which our equilibrium analysis does not address.

Furthermore, we reduce the number of variables by assuming perfect energy conversion

$a_{P}=1$ and $a_{s}=1$, and no loss of carrion due to other processes δ=0.

The non‐trivial equilibrium for all pools of biomass with positive values has the following expressions for $\hat{R}$, $\hat{C}$, $\hat{P}$, and $\hat{S}$

(A1) $$\hat{R}=\frac{m_{s}q_{P}‐m_{P}q_{S}}{\alpha },$$

(A2) $$\hat{C}=\frac{k_{P}m_{S}\phi_{P}‐k_{S}m_{P}\phi_{S}}{‐\alpha },$$

(A3) $$\hat{P}=\frac{\gamma \left(‐k_{P}m_{S}\phi_{P}q_{S}+k_{S}\left(m_{S}\left(‐1+\phi_{S}\right)q_{P}+m_{P}q_{S}\right)\right)}{\beta },$$

(A4) $$\hat{S}=\frac{\gamma \left(k_{S}m_{P}\phi_{S}q_{P}‐k_{P}\left(m_{S}q_{P}+m_{P}\left(‐1+\phi_{P}\right)q_{S}\right)\right)}{\beta },$$

where

(A5) $$\alpha =k_{S}\phi_{S}q_{P}‐k_{P}\phi_{P}q_{S},$$

(A6) $$\beta =\left(k_{S}m_{S}‐k_{P}m_{P}\right)\left(‐m_{S}q_{P}+m_{P}q_{S}\right)\alpha$$

(A7) $$\gamma =‐vm_{S}q_{P}+Ik_{S}\phi_{S}q_{P}+vm_{P}q_{S}‐Ik_{P}\phi_{P}q_{S}.$$

We tested the local stability as determined by the Jacobian matrix evaluated at the equilibrium

$$\hat{J}=\left(\begin{array}{cccc} ‐v‐k_{P}\hat{P}‐k_{S}\hat{S} & 0 & ‐k_{P}\hat{R} & ‐k_{S}\hat{R}\\ k_{P}(1‐\phi_{P})\hat{P}+k_{S}(1‐\phi_{S})\hat{S} & ‐q_{P}\hat{P}‐q_{S}\hat{S} & q_{P}\hat{C}+k_{P}(1‐\phi_{P})\hat{R} & q_{S}\hat{C}+k_{S}(1‐\phi_{S})\hat{R}\\ k_{P}\phi_{P}\hat{P} & q_{P}\hat{P} & ‐m_{P}+q_{P}\hat{C}+k_{P}\phi \hat{P}\hat{R} & 0\\ k_{S}\phi_{S}\hat{S} & q_{S}\hat{S} & 0 & ‐m_{S}+q_{S}\hat{C}+k_{S}\phi S\hat{R} \end{array}\right)$$

to calculate the eigenvalues, which we evaluated numerically to check that all eigenvalues are negative, indicating a stable equilibrium.

### A.2 Coexistence in model with full population dynamics

For the model with both predators and scavengers, we find the possibility for coexistence of these two consumer types over some range of parameters. Topologically, the model resembles a standard two resources competition model with predators specialized on live prey and scavengers specialized on carrion, so that based on prior theory we could expect coexistence to be one possible outcome depending on conditions (Hulot & Loreau, 2006; Tilman, 1982). However, there are several important differences. First, the resource live prey goes into the other resource pool, carrion. Usually, this requires predators to do this in this model so predators in a way both facilitate and compete with scavengers. Both predators and scavengers are allowed to switch between live prey and carrion, although each switches at different densities according to the preferences of each.

We use mutual invasibility criteria, where each species has to be able to, from a low density, invade a monoculture of the other species for coexistence to occur (Litchman & Klausmeier, 2001; Smith & Waltman, 1995). For the scavenger to invade the system with the predator present, it is a requirement that $\frac{\mathrm{dS}}{\mathrm{Sdt}}>0$. For this to be true (and following our assumption in the main text results that the scavenger does not contribute to the carrion pool $\phi_{S}=1$ and conversion efficiencies $a_{P}=1$ and $a_{s}=1$), the following inequality must be true

(A8) $$\frac{\hat{R}f_{S}+s_{S}{\hat{C}}_{P}}{1+f_{S}\hat{R}h_{\mathrm{RS}}+s_{S}{\hat{C}}_{P}h_{\mathrm{CS}}}>m_{S}$$

where $\hat{R}$, $\hat{C}$, and $\hat{P}$ are defined by the equilibrium of the full model with only the predator present

(A9) $$\hat{R}=\frac{m_{P}}{f_{P}\left(1‐h_{\mathrm{RP}}m_{P}‐h_{\mathrm{CP}}m_{P}\left(1‐\phi_{P}\right)\right)},$$

(A10) $$\hat{C}=\frac{m_{P}\left(1‐\phi \right)}{s_{P}\left(1‐h_{\mathrm{RP}}m_{P}‐h_{\mathrm{CP}}m_{P}\left(1‐\phi_{P}\right)\right)},$$

(A11) $$\hat{P}=\frac{I}{m_{P}}‐\frac{v}{f_{P}\left(1‐h_{\mathrm{RP}}m_{P}‐h_{\mathrm{CP}}m_{P}\left(1‐\phi_{P}\right)\right)}.$$

We can plot the inequality described by Equation (A8) as a region of parameter space defining where the scavenger can invade the system in Figure A1 (shaded region is positive invasion).

Similarly, we can define when the predator can invade a system at equilibrium with only the scavenger present so that $\frac{\mathrm{dP}}{\mathrm{Pdt}}>0$ as a region of parameter space as depicted in Figure A2 (shaded region is positive invasion).

We find that for the range of parameters where we have positive biomass of both predators and scavengers as depicted in Figure A3, the mutual invasibility criteria ($\frac{\mathrm{dS}}{\mathrm{Sdt}}>0$ and $\frac{\mathrm{dP}}{\mathrm{Pdt}}>0$) are satisfied; thus, stable coexistence is possible in the Full model.

### A.3 Additional calculations

#### A.3.1 Parameter values

Calculation of $n_{P,S}$:

The interference competition parameter, $n_{P,S}$, affects handling time and thus kill interval, the sum of handling time of current prey and time spent searching and killing next prey (Tallian et al., 2017). The effect of bears on lynx has been found to result in 1.5 days shorter feeding (handling) time (Krofel et al., 2012). The effect of bears on wolves has been found to result in 7.6 hr longer kill interval (handling) time (Tallian et al., 2017). Assuming a kill interval of 2–3 days (Tallian et al., 2017), the maximum impact on handling time is thus a 75% decrease or increase so we present results where we have constrained $n_{P,S}$ to be between −1 and 1.

Calculation of $\phi_{P,S}$:

The proportion a predator immediately consumes, $\phi_{P,S}$, varies with context. It is possible that a predator kills a prey and does not consume any of the carcass ($\phi_{P,S}=0$). Typically however, some proportion is consumed and that proportion consumed varies between species. For example, a minimum predator consumption based on metabolic requirements has been calculated for a lynx to be 1.7 kg (Andrén et al., 2011), for a wolverine to be 1.2 kg (Andrén et al., 2011), and for a wolf to be 3.25 kg (Wikenros et al., 2013). If we consider an average reindeer mass to be 32 kg, then percent immediately consumed based on metabolic requirements for a wolf is about 10%, lynx is 5% and wolverine 4% of the carcass biomass. Lynx‐killed reindeer are rarely consumed entirely but up to 20%–90% can be consumed as lynx use reindeer carcasses for an average of two to three nights and may consume about 2.5 kg per night (Pedersen et al., 1999). A wolverine may consume up to 70 % of its kill (Andrén et al., 2011). Andrén et al. (2011) estimate a value of 41% for lynx consumption of slaughter weight of a reindeer since lynx do not usually consume all edible parts. Thus, we focus on values between 0.4 and 0.5 but also consider a range of 0 to 1 for $\phi_{P,S}$.

Calculation of $z_{P,S}$:

The scaling parameter, $z_{P,S}$, affects the relatively fixed propensity to scavenge and may vary between species. Although we generally assume that $z_{P}>z_{S}$, we also explore conditions where $z_{P}=z_{S}=1$. For the wolverine, the majority (61%) of its food can come from scavenging lynx‐killed reindeer (Mattisson, Andrén, et al., 2011), while for some species we assume this value can be much less so we use the range of 0 to 1 for $z_{P,S}$.

#### A.3.2 Other metrics of predation

We also calculate several other metrics of predation and report the results in the sections that follow for each model. Prey killed per predator is total predation per predator:

(A12) $${\mathrm{Kills}}_{\mathrm{Total}}/\mathrm{predators}=k_{P}+\frac{k_{S}S}{P}.$$

Per‐capita prey killed per predator is total predation per prey per predator:

(A13) $${\mathrm{Kills}}_{\mathrm{Total}}/\mathrm{prey}/\mathrm{predators}=\left(k_{P}P+k_{S}S\right)/R/P.$$

Per‐capita prey killed by predators per predator is predator kill rate per prey:

(A14) $$\mathrm{Kill}\;\mathrm{rat}e_{\mathrm{Predator}}/\mathrm{prey}=k_{P}/\mathrm{R.}$$

### A.4 Additional results

#### A.4.1 Model with full dynamics

In this model, we generally used a fixed prey resource input (Jansen & Van Gorder, 2018), g(R)=I‐vR, to simplify the model and speed up simulations (Figure A4).

For this model with full dynamics:

We now look at predation metrics within the range of parameters where the predators and scavengers coexist to compare to the other models where both guilds are present:

Total predation is relatively constant with increases in scavengers (Figure 4a). Positive $n_{P}$ (increase in handling time with scavenger abundance) can have a small negative effect on total predation, and negative $n_{P}$ (decrease in handling time with scavenger abundance) can have a small positive effect on total predation. Although total predation is relatively constant, there is a very slight decline with increases in scavengers, and this decline depends on v, the prey efflux rate from the system. Larger values of v create larger declines. As v approaches I, the prey influx, total predation can become more nonlinear and have a more pronounced decline with increases in scavengers.

Predator kill rate increases with scavengers (Figure 4d). Positive $n_{P}$ (increase in handling time with scavenger abundance) can have a small positive effect on predator kill rate, while negative $n_{P}$ (decrease in handling time with scavenger abundance) can have a small negative effect on predator kill rate. What is interesting is that the “+” is on the upside part of line, meaning increased handling time actually increases kill rate. However, prey have higher abundance and predators have lower abundance with increased handling time so this makes sense.

Prey killed per predator increases with scavengers (Figure A5). Positive $n_{P}$ (increase in handling time with scavenger abundance) can have a small positive effect on predation per predator, while negative $n_{P}$ (decrease in handling time with scavenger abundance) can have a small negative effect on predation per predator. Per capita prey killed per predator decreases and then increases with scavengers (Figure A5). Positive $n_{P}$ (increase in handling time with scavenger abundance) makes per capita prey killed per predator decrease and then increase even more with scavengers, while negative $n_{P}$ (decrease in handling time with scavenger abundance) makes per capita prey killed per predator decrease and then increase less with scavengers. Per capita prey killed by predators per predator decreases with scavengers (Figure A5).

### A.4.2 Model with only R and C dynamics

In this model, we only use Equations 5 and 6, setting predator and scavenger populations to constant values. We used a nonlinear growth rate for the prey where the input of the prey to the system is defined as g(R)=Rμ(1‐R/K), however, we find the same qualitative results when we use a fixed prey resource input, g(R)=I‐vR. For this model with only R and C dynamics (Figure A6):

Total predation increases with scavengers (Figure 4b). Predator kill rate increases and then decreases with scavengers (Figure 4e). There is a small effect of $n_{P}$ (change in handling time with scavenger abundance). Prey killed per predator increases with scavengers (Figure A7). Remember that the number of predators remains the same in this model. Per‐capita prey killed per predator increases with scavengers (Figure A7). Per‐capita prey killed by predators per predator increases with scavengers (Figure A7).

### A.4.3 Model with no dynamics

For this model with no dynamics:

Total predation increases with scavengers (Figure 4c). Predator kill rate is relatively constant with scavengers (Figure 4f). However, positive $n_{P}$ (increase in handling time with scavenger abundance) can have a small negative effect on predator kill rate, while negative $n_{P}$ (decrease in handling time with scavenger abundance) can have a small positive effect on predator kill rate. Prey killed per predator increases with scavengers (Figure A8). This is because number of predators remains the same in this model. Per‐capita prey killed per predator increases with scavengers (Figure A8). Per‐capita prey killed by predators per predator is constant with scavengers (Figure A8) with a similar small effect of $n_{P}$ on predator kill rate.

In this simple model, R and C are constant, as is $h_{\mathrm{RP}}$. Recall the assumption that predation propensity f=1‐s, thus predator kill rate is proportional to $\frac{1‐s_{P}}{2+s_{P}\left(h_{\mathrm{CP}}‐1\right)}$. Therefore kill rate is a function of scavenging propensity by the predator $s_{P}$ and handling time of the carrion by the predator $h_{\mathrm{CP}}$. If $h_{\mathrm{CP}}$ is not in the equation for $s_{P}$, increasing handling time $h_{\mathrm{CP}}$ always decreases kill rate as stated in the Section 3. With this assumption, if interference competition $n_{P}=0$, scavenger density S has no effect on predator kill rate. However, a positive value for $n_{P}$ increases handling time, and thus increases in S decreases predator kill rate. A negative value for $n_{P}$ decreases handling time, and thus increases in S increases predator kill rate.

We include handling time of the carrion by the predator $h_{\mathrm{CP}}$ in the equation for scavenging propensity by the predator $s_{P}$ though because we expect predators to generally behave adaptively (Hilborn et al., 2018). Recall the equation for $s_{P}\left(C\right)=\frac{z_{P}C^{b_{P}}}{1+z_{P}h_{\mathrm{CP}}C^{b_{P}}}$, thus handling time of the predator affects the scavenging rate, which, also affects kill rate. For the moment let us assume that scavenging propensity scaling coefficients $z_{P}=1$ and $b_{P}=0$. Then a positive value for $n_{P}$ means that S increases predator handling time, and thus predator scavenging rate decreases. A negative value for $n_{P}$ decreases handling time, and thus scavenging rate increases. It can be easily shown that predator kill rate is proportional to $\frac{1}{1+s_{P}h_{\mathrm{CP}}}$. Therefore, because S affects both $s_{P}$ and $h_{\mathrm{CP}}$, it is not immediately obvious if increasing S will increase or decrease kill rate. However, it can be shown that under these assumptions, $h_{\mathrm{CP}}$ is more strongly affected than $s_{P}$ by S so increases in S decreases predator kill rate for $n_{P}>0$ if the predator strategy in scavenging behavior is adaptive to S density. Note this is the same result as above when the predator strategy in scavenging behavior is non‐adaptive (does not affect decision) in response to S density so we can conclude that, under these assumptions, S decreases predator kill rate if it increases predator handling time.

For other assumptions on scavenging propensity scaling coefficients z, b, and carrion C, there are certain combinations of values that preserve this relationship of S always decreases predator kill rate for $n_{P}>0$ and increases predator kill rate for $n_{P}<0$ (see Appendix Section A.7). This is the result of handling time being in both the equation for scavenging propensity s and in the denominator in the functional response.

### A.5 Parameters that model more symmetry in the interactions between predator and scavenger

Here, we allow the scavenger to contribute to the carrion pool by setting $\phi_{S}>0$. This is likely important in the interaction between some species such as the lion and hyena. We also consider interference competition by the predator on the scavenger $n_{S}\neq 0$ for added realism of the interaction between species such as the lion and hyena.

These assumptions do make small quantitative differences in the predation patterns we observe, but we do not find any qualitative differences, for example, in comparing the following figures (Figure A9) with the figures in the main text for the full model (Figure 4). For the R and C dynamics model, we also found no qualitative differences with these assumptions.

### A.6 Loss processes

Here, we relax our earlier simplifying assumptions and allow loss processes to occur to the carrion pool by setting δ>0, and loss processes on energy conversion by setting both $a_{P}$ and $a_{S}$ to be <1. These parameters not only influence pool sizes, especially those of predators and scavengers, but can alter rates due to all of the behavioral, consumption, and population growth processes and feedbacks in the Full dynamics model (Figures A10, A11). However, changes in these parameters generally have a small quantitative effect but not much qualitative effect on our metrics. In the Full dynamics model, different values of energetic conversion factors $a_{P}$ and $a_{S}$ can lead to a reduced or slightly different coexistence region of parameter space for predators and scavengers. However, we do not assume the conversion factors differ between predators and scavengers, nor do we assume that it differs between prey and carrion. Note that these conversion parameters $a_{P}$ and $a_{S}$ do not exist in the two simpler models because predators and scavengers are constrained to be constant in those models.

### A.7 **Form of scavenging** s **equation**

Here, we investigate the impact of the form of the scavenging s equation if we consider other values of scavenging propensity scaling coefficients z, b, and carrion C. We know that if we remove handling time h from the scavenging s equation so we only have h in the denominator of kill rate, then kill rate is negatively related to handling time (Section 3 in main text and Appendix Section A.4.3). However, there is a parameter range that allows us to change the handling time and keep the relationship intact of kill rate negatively related to handling time as can be seen in the following figures in the blue regions. Our results hold for when this condition is satisfied or any of the previous simplified conditions are satisfied (Appendix Section A.4.3). Fryxell and Lundberg (1994) have handling time in the preference and the functional response and the step function that resembles ours. They also have a z parameter that dictates the closeness of diet choice to optimal step function, which is what our b exponent does (Figures A12 and A13).
